# Anaerobic flora, *Selenomonas ruminis* sp. nov., and the bacteriocinogenic *Ligilactobacillus salivarius* strain MP3 from crossbred-lactating goats

**DOI:** 10.1038/s41598-024-54686-6

**Published:** 2024-02-28

**Authors:** Saranporn Poothong, Somboon Tanasupawat, Somchai Chanpongsang, Engkarat Kingkaew, Chackrit Nuengjamnong

**Affiliations:** 1 Department of Animal Husbandry, Faculty of Veterinary Science, Chulalongkorn University, Bangkok, 10330 Thailand; 2 Department of Biochemistry and Microbiology, Faculty of Pharmaceutical Sciences, Chulalongkorn University, Bangkok, 10330 Thailand; 3 Department of Biology, School of Sciences, King Mongkut’s Institute of Technology Ladkrabang, Bangkok, 10520 Thailand; 4 Center of Excellence for Food and Water Risk Analysis (FAWRA), Faculty of Veterinary Science, Chulalongkorn University, Bangkok, 10330 Thailand

**Keywords:** Computational biology and bioinformatics, Microbiology, Molecular biology

## Abstract

This study aimed to examine the distribution of anaerobic bacteria in the rumen fluid of Thai crossbred goats and to screen potential probiotic strains capable of producing antimicrobial compounds and inhibiting bacteria that cause milk fat depression. Thirty-four strains of bacteria from the rumen fluid were divided into 13 groups within 12 genera based on 16S rRNA gene sequences. The RF1-5 and RF5-12 were identified as *Streptococcus luteliensis* and *Bacillus licheniformis*, respectively, and demonstrated non-ropy exopolysaccharide. Furthermore, mPRGC5^T^ was closely related to *Selenomonas caprae* JCM 33725^ T^ (97.8% similarity) based on 16S rRNA gene sequences. It exhibited low average nucleotide identity, digital DNA–DNA hybridization, and average amino acid identity values with related type strains ranging from 84.9 to 86.0%, 21.3 to 21.8%, and 73.8 to 76.1%, respectively. The genotypic and phenotypic characteristics of mPRGC5^T^ strongly support this strain as a new species of the genus *Selenomonas* for which the name *Selenomonas ruminis* mPRGC5^T^ was proposed. The type strain is mPRGC5^T^ (= JCM 33724^ T^ = KCTC 25177^ T^). *Ligilactobacillus salivarius* MP3 showed antibacterial activity against *Cutibacterium acnes* subsp. *acnes* DSM 1897^ T^ and *Kocuria rhizophila* MIII. The enterolysin A cluster gene was identified in its genome. The auto-aggregation of *L. salivarius* MP3 was 93.6 ± 0.2%. Additionally, co-aggregation of *L. salivarius* MP3 with *C. acnes* DSM 1897^ T^ and *K. rhizophila* MIII had 92.2 ± 3.4% and 87.3 ± 4.5%, respectively. The adhesion capacity of strain MP3 was 76.11 ± 2.2%. Probiogenomic analysis revealed that *L. salivarius* MP3 was nonhazardous to animal supplementation and included acid- and bile-tolerant ability. However, strain MP3 contained three antibiotic resistance genes. Thus, the supplementation of *L. salivarius* MP3 could increase the milk fat content by suppressing *C. acnes* DSM 1897^ T^ with antibiotic resistance gene horizontal transfer awareness.

## Introduction

Ruminants convert human indigestible plant material into high-nutritional-value meat and dairy products. Milk contains protein and fat, which are highly valuable components. Milk fat depression (MFD) is a metabolic syndrome that has been prevalent for several decades and is characterized by a consistent decrease in the amount of milk fat. The impact of dietary changes on milk fat is significant, and this topic has been extensively investigated in ruminant animals^1^. The main hypothesis for the incidence of strong MFD involves a transition in the biohydrogenation process. In vitro studies have demonstrated that *Cutibacterium acnes* involved in biohydrogenation process by biosynthesize *trans*-10, *cis*-12 conjugated linoleic acid from linoleic acid. Additionally, *C. acnes* is the predominant species associated with the *trans*-10-shift phenomenon the cause MFD in vivo study^2^. However, the antibacterial effects against bacteria causing milk fat depression have not been investigated.

The rumen is a vital digestive organ in ruminants and is a habitat for one of the most complex microecosystems. Generally, abundant lactobacilli and streptococci in the rumen are the most common bacteria in sucking calves and lactating ruminants fed a high-concentrate diet. The predominant lactic acid bacteria (LAB) in the rumen are *Lactobacillus ruminis, L. acidophilus, L. fermentum, L. helveticus, Streptococcus bovis, S. faecalis, S. faecium, S. equinus, Bifidobacterium globosum, B. longum, B. adolescentis and B. ruminale*^3^. These bacteria adhered to the rumen epithelium and released organic acids that were desirable to the host. *Streptococcus bovis* rapidly fermented starch and produced lactic acid as an end-product. Additionally, *Selenomonas ruminantium* hydrolyzed starch and released lactic acid and propionic acid as fermentation products^4^.

Lactic acid bacteria were distributed in the rumen and used as probiotics in ruminants. LAB synthesized the potentially antimicrobial compounds, and they had probiotic effects. Antimicrobial metabolites of LAB included lactic acid, acetic acid, ethanol, hydrogen peroxide, bacteriocins, antifungal peptides, and exopolysaccharides. Organic acid inhibited pathogenic microbes by reducing the pH of the habitat. The bactericidal effect of hydrogen peroxide varied with concentration and environmental conditions^5^. Furthermore, the bacteriocins and beneficial metabolites produced by LAB have been commercially utilized^6^. Bacteriocins comprise a broad and narrow spectrum. It can cause bactericidal or bacteriostatic effects. Bacteriocins were categorized into three groups (Class I, Class II, and Class III) based on their post-translational modification, enzyme stability, and thermal stability. However, research on class III bacteriocins is limited. Moreover, the probiotic activity of LAB bacteriocin-producing strains played a vital role in preventing pathogenesis in animals and improving growth performance and production^7^. Therefore, the most effective probiotic microorganisms were native inhabitants of the target animals. The probiotic candidates were nontoxic, nonpathogenic, and resistant to the pH, temperature, and bile salts in the gastrointestinal tract. Notably, potential probiotics must be antimicrobial, adhere to the intestinal mucosa, modulate the immune system, and be genetically stable. Consequently, when searching for wild-type probiotic candidates, it is preferable to obtain bacterial strains from the microorganism's natural environment in the gastrointestinal tract of the host^8^.

Therefore, this study investigated the distribution of anaerobic bacteria in the rumen fluid of Thai crossbred goats and screened putative probiotic bacteria to produce antimicrobial substances and inhibit milk fat-depression bacteria.

## Results and discussion

### Identification of bacterial isolates

**Figure 1:**
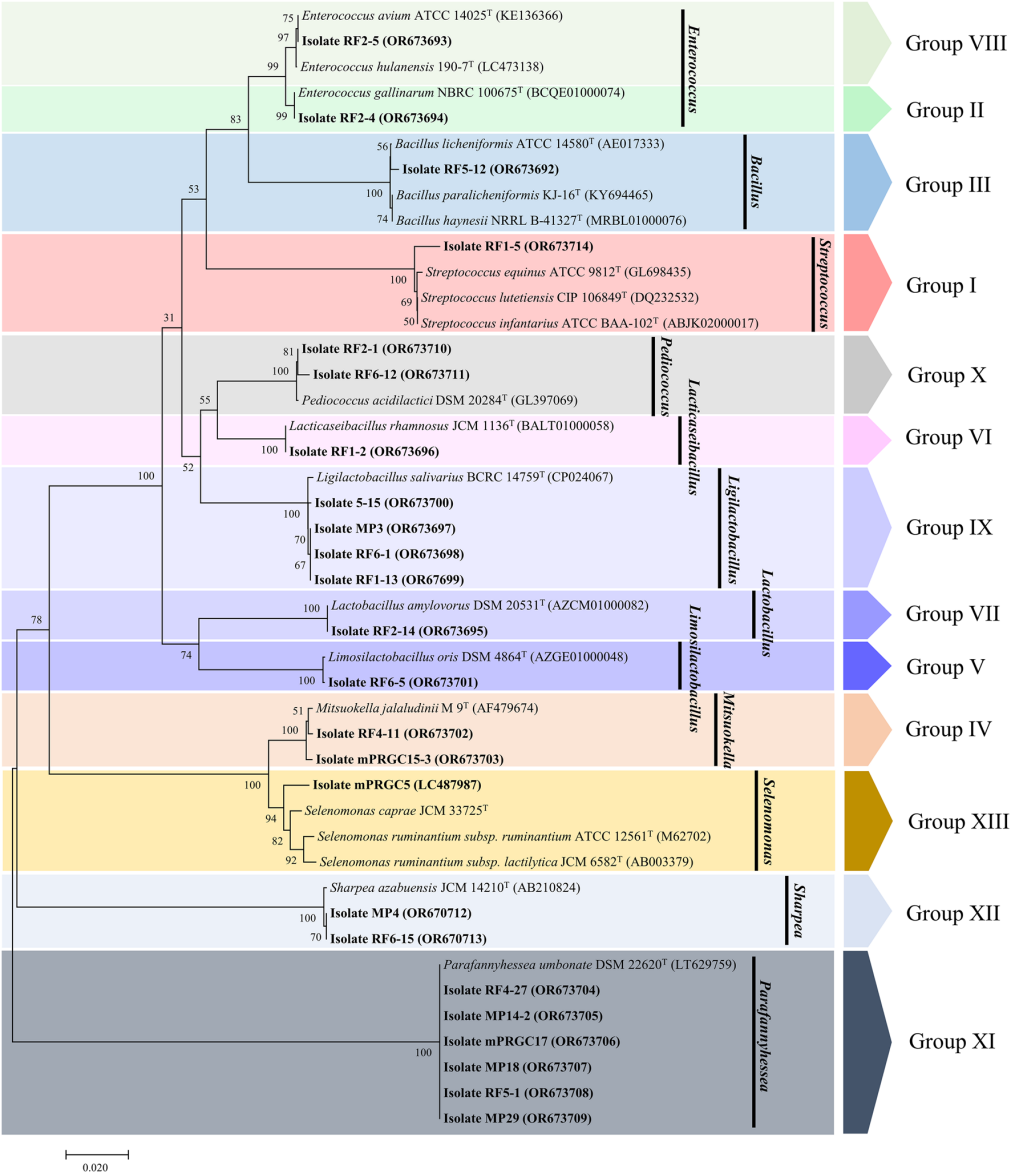
The neighbor‒joining phylogenetic tree of representative strains of thirteen groups based on the 16S rRNA gene divided by dendrogram analysis.

Thirty-four bacterial strains were isolated from rumen fluid samples of Thai crossbred Saanen lactating goats (Table S1). Based on phenotypic characteristics and 16S rRNA gene sequencing, the hierarchical cluster divided all the strains into 13 groups (Figs. 1, S1). The different phenotypes presented different cell morphologies, Gram staining, catalases, biochemical activities, and bacterial oxygen requirements. The genera of all the strains consisted of *Bacillus, Enterococcus, Lactobacillus, Lacticaseibacillus, Ligilactobacillus, Limosilactobacillus, Mitsuokella, Parafannyhessea, Pediococcus, Selenomonas, Sharpea* and *Streptococcus*. The 29 strains were Gram-positive and negative for catalase. The two strains were Gram-positive and positive for catalase, and the other two strains were Gram-negative and catalase positive. Finally, only one curved rod shape was negative for both the Gram stain and the catalase enzyme (Table S2).

Group I consisted of two cocci strains (RF1-5 and RF1-10). These bacteria were cultured in the medium pH 9, 3% NaCl concentration, and at a temperature of 45 °C. This group was facultative anaerobic bacteria. Arginine hydrolysis and nitrate reduction were positive. Partial hemolysis was displayed. The end products of glucose fermentation included L-lactic acid and acetic acid. This group was identified as *Streptococcus luteliensis* (similarity 99.01%).

Group II included one Gram-positive coccus (RF2-4). The strain was grown at pH 3, 15 ℃ and 45 ℃. The hazy zone was shown in the hemolysis test. The other phenotypic characteristics were presented in Table S2. Lactic acid and acetic acid were products of glucose fermentation. The strain RF2-4 showed the highest 16S rRNA gene sequence similarity to *Enterococcus gallinarum* NBRC 100675^T^.

Group III included two rod-shaped strains (RF4-12 and RF5-12) that were Gram-positive. These rods grew over a wide pH range from 3 to 9 and at various temperatures (15 °C and 45 °C). The strains produced lactate, acetate, and propionate. Based on the results of the 16S rRNA gene sequence comparison (99.7% similarity), these strains were identified as *Bacillus licheniformis*.

Group IV included two strains of strictly anaerobic, Gram-negative bacilli (RF4-11, mPRGC15-3). These strains exhibited alpha hemolysis. Two bacilli were able to proliferate at 45 °C and pH 3, 9. The end products of glucose fermentation included lactate, acetate, and propionate. Two strains were identified as *Mitsuokella jalaludinii*.

Group V (RF6-5, RF2-23) included two Gram-positive, rod-shaped strains that were grown in media with a pH range of 3–9 and proliferated at 45 °C. Lactate and volatile fatty acids (acetate and propionate) were the main products. Based on 16S rRNA analysis, this group was identified as *Limosilactobacillus oris* (Table S1).

Group VI consisted of strain RF1-2, which was closely related to *Lacticaseibacillus rhamnosus* JCM 1136^ T^. The cells were Gram-positive and catalase-positive. The strain RF1-2 was grown in media with a pH ranging from 3 to 9 and 3% NaCl. Furthermore, the strain was grown at different temperatures from 15 to 45 °C. Arginine hydrolysis was negative when nitrate reduction was positive. The two main products of glucose fermentation were lactic acid and acetic acid.

Group VII only contained strain RF2-14. This strain exhibited a rod shape and was Gram-positive. The phenotypes presented the survival at pH 3 and 45 °C. Arginine and nitrate were both negative. Lactate and acetate were the main products of glucose utilization. The strain RF2-14 was identified as *Lactobacillus amylovorus* using 16S rRNA sequencing, which showed a similarity of 99.8%.

Group VIII contained two cocci strains (RF1-4 and RF2-5). Both strains grew at wide-range temperatures ranging from 15 to 45 °C. The hemolysis results revealed alpha hemolysis. The ability to use the various carbohydrates and end products from glucose fermentation was presented in Table S2. The RF2-5 strain, representative of this group, showed 99.7% similarity with *Enterococcus avium* ATCC 14025^T^.

In Group IX, seven strains (MP3, RF3-6, RF6-1, RF6-11, RF2-22, RF1-13, and RF5-15) exhibited Gram-positive and negative results for catalase production, gas production, arginine hydrolysis, and nitrate reduction. All seven strains exhibited partial hemolysis. These bacteria produced L-lactic acid and acetic acid. However, two strains from this group synthesized propionic acid and butyric acid from glucose fermentation, as shown in Table S2. These strains were identified as *Ligilactobacillus salivarius* based on 16S rRNA similarity (99.78–99.80%).

Group X included two facultative tetracoccal strains (RF6-12 and RF2-1). Two strains survived in the pH range from 3 to 9 and temperature range from 15 to 50 °C. These two strains converted glucose to lactate and acetate. Furthermore, no hemolytic activity was found for either strain. Based on the 16S rRNA gene sequence (99.50–99.83%), these bacteria were identified as *Pediococcus acidilactici*.

Group XI included nine obligately anaerobic strains (RF3-23, RF4-27, MP29, RF5-1, MP20, MP18, mPRGC17, MP16-1 and MP14-2). All the strains could grow at 45 °C and pH 3–9. The strains produced different end products from glucose fermentation in a strain-dependent manner. In addition, strains RF4-27, MP14-2, mPRGC17, MP18, RF5-1, and MP29 were closely related to *Parafannyhessea umbonata* DSM 22620^T^, with 99.9 to 100.0% similarity, respectively.

Group XII included strain MP4 and RF6-15. Both strains were strictly anaerobes. These two rods were distinct from the others because they generated gas products from glucose fermentation. Similarly, lactate and acetate were the major products of glucose utilization. The two strains were able to proliferate at pH 3, pH 9, 15–45 °C, and 3% NaCl. No positive hemolysis was found. MP4 and RF6-15 were identified as *Sharpea azabuensis* based on the 16S rRNA gene sequence (99.7% similarity).

Group XIII included the curved-rod anaerobic bacterium (strain mPRGC5). This strain exhibited different phenotypes from the other strains (Table S2). The strain mPRGC5 was grown in the presence of L-arabinose, D-cellobiose, fructose, D-galactose, D-glucose, lactose, D-mannose, D-maltose, salicin, sucrose, and D-xylose. Lactate, acetate, and propionate were produced from glucose fermentation. Based on the16S rRNA gene sequence (97.88% similarity, 1407 bp) with *Selenomonas caprae* JCM 33724^T^, it was lower than the species delineation value. Hence, this strain could be a novel species of the genus *Selenomonas*.

The distribution of LAB in the rumen was similar to that in the dairy bovine rumen according to a previous study. According to Hu, et al.^9^, the study isolated potential LAB probiotics from rumen fluid, which included *Enterococcus avium*, *Streptococcus lutetiensis*, and *Streptococcus equinus*. These species were identical to those found in this study. In addition, another study identified the LAB isolated from the rumen fluid and feces of dairy cows as *Lactiplantibacillus plantarum*, *Ligilactobacillus salivarius*, and *Limonilactabacillus fermentum*^10^. *L. amylovorus*, *L. rhamnosus*, *L. salivarius*, *L. oris*, and *P. acidilactici* were identified as members of the family *Lactobacillaceae* by the LAB strains in the present study. Therefore, the family *Lactobacillaceae* was the dominant LAB isolated from the rumen fluid. The feed composition is an additional variable that can alter the rumen microbiome. According to Bo Trabi, et al.^11^, compared with the low-grain TMR diet, the high-grain TMR diet fed to fattening lambs increased the number of simple sugar-fermentative bacteria, such as *Dialister, Megasphaera, Parafannyhessea,* and *Sharpea*. Similarly, the donors in this study were fed a high-grain TMR diet (60% concentrate). Additionally, a review of ruminomics data from 1,000 cows revealed the core rumen isolates, including *Treponema bryantii* for cellulose degradation, *Prevotella* for hemicellulose degradation, *Parafannyhessea umbonata* for soluble sugar utilization and *Selenomonas ruminantium* for secondary fermentation product utilization^12^. The distributions of *Parafannyhessea* sp. and *Selenomonas* sp. in the rumen fluid of goats were corroborated by a previous study. Furthermore, *Mitsuokella jalaludinii* was found primarily in the rumen of Malaysian cattle, and the bacteria produced phytase, an enzyme commonly used in poultry production^13^. In this study, two isolates of *M. jalaludinii* were isolated from the rumen of goats, confirming the habitat of these bacteria in the rumen. Thus, the rumen of Thai crossbred lactating goats is a valuable isolation source of LAB. However, the bacterial diversity was dependent on the type of donor diet.

### Characterization of novel obligate anaerobic *Selenomonas* species

**Figure 2:**
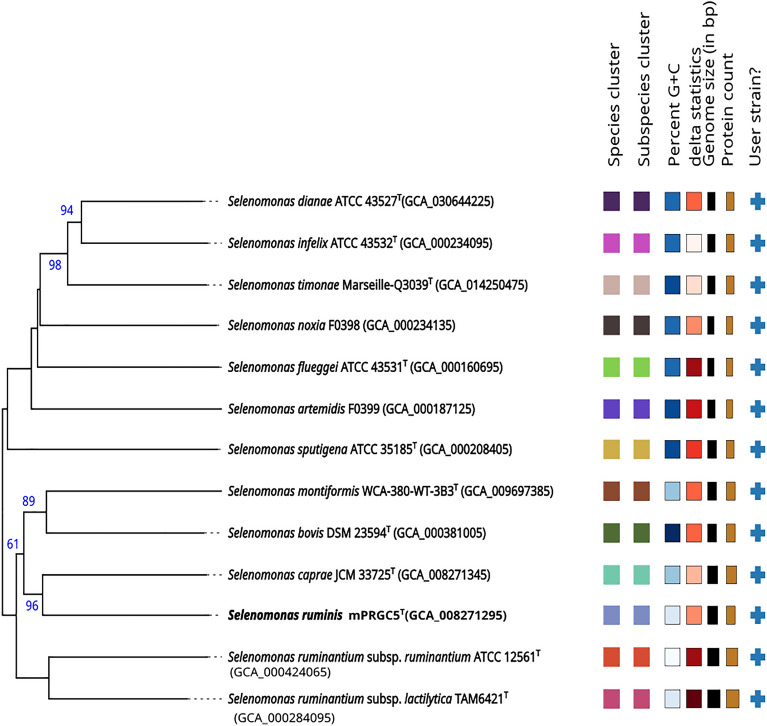
The phylogenomic tree of the mPRGC5^T^ strain and its closely related strains was reconstructed via TYGS.

According to 16S rRNA gene sequencing, the strain belongs to the family *Selenomonadaceae* (phylum Firmicutes). The closely related species were *Selenomonas caprae* JCM 33725^T^ (97.88% similarity; 27 nt difference at 1275), *Selenomonas ruminantium* subsp*. ruminantium* ATCC 12561^T^ (96.49% similarity; 49 nt difference at 1395) and *Selenomonas ruminantium* subsp. *lactilytica* JCM 6582^T^ (96.39% similarity; 51 nt difference at 1401). A neighbor-joining tree analysis based on the 16S rRNA gene confirmed that mPRGC5^T^ formed a substantial clade with *S. caprae* JCM 33725^T^, *S. ruminantium* subsp*. ruminantium* ATCC 12561^T^ and *S. ruminantium* subsp. *lactilytica* JCM 6582^T^, which were originally from a similar environment. The phylogenomic tree also confirmed that mPRGC5^T^ was related to *S. caprae* JCM 33725^T^ (Fig. 2). The draft genome sequence of mPRGC5^T^ was obtained from this study. The ANIm, ANIb, dDDH and AAI values are shown in Table S3. The draft genome data revealed the closest phylogenetic relatives to *S. caprae* JCM 33725^T^ (ANIb 79.43%, ANIm 86.16%, dDDH 23.50%, AAI 82.6%), *S. ruminantium* subsp. *lactilytica* JCM 6582^T^ (ANIb 73.80%, ANIm 86.17%, dDDH 20.40%, AAI 73.6%) and *S. ruminantium* subsp. *ruminantium* DSM 2150^T^ (ANIb 74.25%, ANIm 84.47%, dDDH 21.2%, AAI 73.3%). All ANI and AAI values were less than the 95–96% delineation cut-off. Additionally, the dDDH levels obtained in this study were less than 70%, which suggested that the strain under investigation belongs to a newly unidentified species. Consequently, strain mPRGC5^T^ can be classified as a novel species within the genus *Selenomonas*.

The strain mPRGC5^T^ was found to grow well on PYG agar. This strain was grown under strictly anaerobic conditions. After 2 days of incubation, the colonies were white, circular, convex, and opaque (1.0–3.0 mm in diameter). The cells exhibited curved rods (Fig. S2), which were characterized by their gram-negative and nonspore-forming ability. The dimensions of these cells ranged from 1.6 to 3.8 µm in length and 0.4–0.5 µm in width. Strain mPRGC5^T^ exhibited the motile peritrichous flagella required for locomotion. The identification of the flagellar assembly proteins *MotB, FliM/N/R/E/I, FlaA, FlgB/C/D/H, FleN,* and *FlhA/B/F* also confirmed the presence of flagella. The different phenotypic characteristics of strain mPRGC5^T^ and closely related type strains are described in Table 1. The strain mPRGC5^T^ was grown at pH 6.0–10.0, 22–39 °C, and 1% (w/v) NaCl. The nitrate reduction, methyl red test, and starch hydrolysis were positive. Contrary, catalase, indole production, hydrogen sulfide production, the Voges–Proskauer test, gelatin liquefaction, and casein hydrolysis were negative. The acid produced from carbohydrate fermentation was shown in Table S4. Similar patterns of carbohydrate utilization were observed between strain mPRGC5^T^ and closely related type strains. In addition, the carbohydrate utilization was further genomically supported. The genomic data of strain mPRGC5^T^ contained the several carbohydrate-associated genes, such as L-arabinose utilization (EC: 5.1.3.4, 2.7.1.16, 5.3.1.4), cellobiose metabolism (EC: 2.7.1.69, 3.2.1.86), fructose utilization (EC: 2.7.3.9, 2.7.1.69, 2.7.1.56), inositol catabolism (EC: 1.1.1.18, 4.2.1.44, 2.7.1.92), lactose and galactose uptake and utilization (EC: 5.1.3.2, 2.7.1.144, 3.2.1.89, 2.7.1.69, 2.7.1.6, 3.2.1.85, 5.3.1.26, 5.1.3.3, 2.7.7.10), maltose and maltodextrin utilization (EC: 2.4.1.25, 3.2.1.10, 2.4.1.1, 3.2.1.20, 5.1.3.3, 3.2.1.20, 2.7.1.69), mannose metabolism (EC: 5.4.2.8, 2.7.1.69, 5.3.1.8, 2.7.7.22), and xylose utilization (EC: 2.7.1.17). This strain demonstrated vigorous acid phosphatase activity and weak alkaline phosphatase, esterase, esterase lipase, and naphthol-AS-BI-phosphohydrolase activity. The cellular fatty acid composition of strain mPRGC5^T^consisted of C_18:1 *ω9c*_ (17.5%), C_16:1 *ω9c*_ (9.1%), C_11:0_ (7.0%), and C_15:1 *ω8c*_ (6.7%) (Table 2). The presence of C_18:1 *ω9c*_ and C_16:1 *ω9c*_ was commonly observed in strain mPRGC5^T^ and in the closely related type strains. The fatty acid C_11:0_ exhibited similarity primarily with *S. ruminantium* subsp. *lactilytica* JCM 6582^T^. Furthermore, strain mPRGC5^T^ contained phosphatidylethanolamine, three unidentified aminophospholipids, an unidentified ninhydrin positive glycolipid, a phospholipid, and two unidentified lipids (Fig. S3). The final products from glucose fermentation were acetate, propionate, valerate, hexanoate, and heptanoate. The strains of *S. caprae* JCM 33725^T^, *S. ruminantium* subsp. *lacticolytica* JCM 6582^T^, and *S. ruminantium* subsp. *ruminantium* DSM 2150^T^ were found to synthesize acetic acid, propionic acid, and valeric acid. In addition, the ability of the mPRGC5^T^ strain to produce *n*-heptanoic acid was similar to that of *S. caprae* JCM 33725^T^.

**Table 1:** Differential characteristics of the mPRGC5^T^ strain and related type strains. Strain 1, mPRGC5^T^; 2, *S. caprae* JCM 33,725^T^; 3, *S. ruminantium* subsp. *lacticolytica* JCM 6582^T^; 4, *S. ruminantium* subsp. *ruminantium* DSM 2150^T^.

Characteristics	1	2	3	4
Colony form	Circular	Irregular	Circular	Irregular
Flagella	Peritrichous	Peritrichous	-	-
pH for growth	6.0–10.0	6.0–9.0	4.0–9.0	4.0–9.0
Temperature (°C)	22–39	20–45	20–48	20–45
Maximum NaCl (%)	1	3	3	1
Starch hydrolysis	+	–	+	–
Acid production from				
Glucose	+	+	+	+
Amidon (starch)	+	–	+	–
Amygdalin	–	w	–	–
D-Adonitol	–	–	–	w
L-Arabinose	+	+	+	w
D-Arabitol	–	–	–	w
D-Celiobiose	+	+	–	+
Erythritol	–	–	–	w
Fructose	+	+	+	+
D-Galactose	+	+	+	+
Glycerol	–	–	–	w
Inositol	+	–	–	+
Inulin	–	–	–	w
D-Mannitol	–	+	w	+
D-Mannose	+	+	w	+
D-Melezitose	–	–	–	w
D-Melibiose	–	+	+	+
D-Raffinose	–	+	+	+
L-Rhamnose	–	–	+	+
D-Ribose	–	–	w	+
Sucrose	+	+	+	+
Salicin	+	+	w	+
D-Sorbitol	–	–	–	w
D-Tagatose	–	–	–	w
D-Trehalose	–	–	–	+
Xylitol	–	–	–	w
D-Xylose	+	+	–	w
DNA G + C content (mol%)	50.06	53.01	50.1	50.1

+ , positive; w, weakly positive; −, negative.

**Table 2:** Cellular fatty acid composition of the mPRGC5^T^ strain and closely related strains.

Fatty acid	1	2	3	4
Straight-chain fatty acids				
C_10:0_	0.9	0.6	–	1.6
C_11:0_	7.0	3.1	7.0	1.7
C_12:0_	5.7	7.9	2.2	4.5
C_13:0_	3.7	3.6	8.7	1.9
C_14:0_	3.1	5.1	1.3	4.5
C_16:0_	4.8	5.4	2.2	8.6
C_17:0_	2.2	1.2	0.8	4.7
C_18:0_	1.1	1.0	0.4	2.0
Unsaturated fatty acids				
C_15:1_ *ω6c*	–	–	0.5	–
C_15:1_ *ω8c*	6.7	6.0	17.7	3.0
C_16:1_ *ω9c*	9.1	19.3	6.5	10.0
C_17:1_ *ω8c*	5.1	–	–	2.7
C_17:1_ *ω9c*	–	4.5	8.0	–
C_18:1_ *ω9c*	17.5	16.8	12.6	27.1
Branched fatty acids				
Iso-C_19:0_	0.8	0.5	–	1.0
C_11:0_ 3OH	0.5	–	–	–
C_12:0_ 3OH	3.4	–	–	–
C_15:0_ 2OH	1.9	0.7	2.1	3.0
C_15:0_ 3OH	0.7	1.1	3.3	1.2
C_16:0_ 2OH	1.9	–	6.3	1.1
Summed features				
1	14.9	8.3	15.1	5.2
2	5.6	11.3	2.5	10.3
3	–	–	–	1.3
8	1.5	1.3	1.0	2.3

Strains: 1, *Selenomonas* mPRGC5^T^; 2, *Selenomonas caprae* JCM 33725^T^; 3, *Selenomonas ruminantium* subsp. *lactilytica* JCM 6582^T^; 4, *Selenomonas ruminantium* subsp. *ruminantium* DSM 2150^T^. The values are percentages of total cellular fatty acids.

−, not present. Fatty acids amounting to less than 0.5% in all the strains were omitted. All the data were analyzed in this study

*Summed feature 1 consisted of *iso*-C_15:1_ H and/or C_13:0_ 3OH.

*Summed feature 2 consisted of a C_12:0_ aldehyde.

*Summed feature 3 consisted of C_16:1_*ω*7*c* and/or C_16:1_*ω*6*c*.

*Summed feature 8 consisted of C_18:1_ *ω*7*c*.

In addition to the results of previous genotypic analysis, mPRGC5^T^ could be distinguished from the closely related species *S. caprae* JCM 33725^T^, *S. ruminantium* subsp. *lactilytica* JCM 6582^T^, and *S. ruminantium* subsp. *ruminantium* DSM 2150^T^ based on its phenotypic characteristics. According to phenotypic characteristics, chemotaxonomic characteristics, in silico G + C content, 16S rRNA gene sequence analysis, ANI, AAI, dDDH, and the genomic annotation findings, the strain mPRGC5^T^ represents a novel species of the genus *Selenomonas*, for which the name *Selenomonas ruminis* sp. nov. is proposed.

## Description of *Selenomonas ruminis* sp. nov.

*Selenomonas ruminis* (ru’mi.nis. L. gen. neut. n. *ruminis*, of the rumen).

The cells were Gram-negative, had peritrichous flagella, and were non-spore-forming, strictly anaerobic bacilli (0.4–0.5 x 1.6–3.8 µm). The colonies were opaque white, circular, convex and 1.0–3.0 mm in diameter. The cells were grown at pH 6.0–10.0 (optimum, pH 7.0), 22–39 ℃ (optimum, 37 ℃) and 1% (w/v) NaCl. Nitrate reduction, methyl red test and starch hydrolysis were positive. Acetate, propionate, valerate, hexanoate and heptanoate are products of glucose fermentation. Acid formation from various carbohydrates included glucose, starch, L-arabinose, D-cellobiose, fructose, D-galactose, inositol, D-lactose, D-maltose, D-mannose, D-sucrose, salicin and D-xylose. Furthermore, the strain mPRGC5^T^ was positive for alkaline phosphatase, esterase, esterase lipase, naphthol-AS-BI-phosphohydrolase and acid phosphatase. The major cellular fatty acids are C_18:1_ ω9c,  C_16:1_ ω9c, C_11:0_ and C_15:1_ ω8c. The polar lipid profile consisted of a phosphatidylethanolamine, three unidentified aminophospholipids, an unidentified ninhydrin-positive glycolipid, a phospholipid and two unidentified lipids. The in silico G + C content is 50.06%. The type strain is mPRGC5^T^ (= JCM 33724^T^, = KCTC 25177^T^, accession no. LC487987, VTOY00000000).

### Volatile fatty acid production

All the strains produced acetic acid as the main product of glucose fermentation (Table 3). Valeric acid is also a common product from all strains except *Ligilactobacillus salivarius* RF6-11, *Parafannyhessea umbonata* MP18, and *Parafannyhessea umbonata* MP20. The VFA results are shown in Table S2. Propionic acid was produced from the bacterial strains RF4-12, MP3, RF6-11, RF6-5, RF4-11, mPRGC15-3, RF3-23, MP14-2, MP16-1, mPRGC17 and MP29. In addition, glucose fermentation revealed that strains MP3, RF6-11, and RF3-23 synthesized butyric acid as their end products. Several strains, including strains RF5-12, MP3, RF6-11, and RF3-23, produced caproic acid. Moreover, strains RF5-12, RF6-11, and RF4-27 demonstrated heptanoic acid production from glucose utilization.

**Table 3:** Volatile fatty acid produced by isolates cultivated in PYG media at 37°C under anaerobic conditions for 48 h.

Isolate no	Acetic acid	Propionic acid	Butyric acid	Valeric acid	Caproic acid	Heptanoic acid
	mmol/µl					
RF5-12	27.77	–	–	0.09	0.38	0.36
RF4-12	11.94	0.94	–	0.18	–	–
RF2-5	6.00	–	–	0.17	–	–
RF1-4	8.41	–	–	0.15	–	–
RF2-4	6.74	–	–	0.16	–	–
RF2-14	5.10	–	–	0.19	–	–
RF1-2	7.09	–	–	0.13	–	–
MP3	33.55	3.06	2.60	2.30	2.56	–
RF3-6	12.51	–	–	0.22	–	–
RF6-1	8.03	–	–	0.14	–	–
RF6-11	45.68	2.90	1.60	–	2.48	2.12
RF2-22	42.05	–	–	0.14	–	–
RF1-13	11.17	–	–	0.13	–	–
RF5-15	13.65	–	–	0.13	–	–
RF6-5	7.79	0.44	–	0.17	–	–
RF2-23	14.68	–	–	0.13	–	–
RF4-11	12.68	0.84	–	0.15	–	–
mPRGC15-3	14.27	1.02	–	0.18	–	–
RF4-27	12.16	–	–	0.12	–	0.16
RF3-23	12.73	1.28	0.54	0.33	1.75	–
MP14-2	12.24	0.93	–	0.18	–	–
MP16-1	13.22	0.95	–	0.16	–	–
mPRGC17	13.59	0.91	–	0.15	–	–
MP18	9.39	–	–	–	–	–
MP20	9.48	–	–	–	–	–
RF5-1	10.92	–	–	0.14	–	–
MP29	9.69	0.54	–	0.17	–	–
RF2-1	6.31	–	–	0.16	–	–
RF6-12	9.94	–	–	0.14	–	–
mPRGC5						
MP4	12.06	–	–	0.37	–	–
RF6-15	11.30	–	–	0.16	–	–
RF1-5	6.50	–	–	0.15	–	–
RF1-10	6.88	–	–	0.13	–	–

−; No detection.

A strictly anaerobic or facultative anaerobic rumen microorganism produces end products consumed directly by the host or indirectly by other microorganisms. VFAs were major microbial end products. Some of these VFAs, such as propionate and butyrate, can be absorbed across the rumen wall and serve as an energy supply for ruminants^14^. In addition, microbial VFAs were considered alternatives to petroleum based VFAs in commercial chemical production due to their sustainability. VFAs have also been used as precursors in anaerobic fermentation to produce biogas and flavor enhancers in the food industry^15^. Furthermore, acetate played a role in the production of terephthalic acid and can also be utilized in polyethylene terephthalate production. Valerate is primarily used in the cosmetics and food and beverage industries. Likewise, the WHO considers valeric acid to be a safe food additive, and it is primarily used as an intermediate substance in the production of flavors and fragrances, agricultural chemicals, and pharmaceuticals^16^. In this study, all the strains isolated from the rumen of Thai goats produced acetic acid through the fermentation of simple sugars. Furthermore, thirty-one strains of bacteria from the goat rumen generate valeric acid as a byproduct of glucose fermentation. Therefore, the goat rumen is presumed to be a beneficial source for identifying potential VFA-producing microorganisms for use in commercial industries and livestock.

### Spot resistant study for antimicrobial activity and exopolysaccharide production

All the strains were screened for antimicrobial activity against the indicator strains. *Ligilactobacillus salivarius* MP3 had inhibitory effects on *Kocuria rhizophila* MIII (55.75 mm in diameter) and *C. acnes* subsp. *acnes* DSM 1897^T^ (30.82 mm in diameter) (Fig. S4). Additionally, *Streptococcus luteliensis* RF1-5 and *Bacillus licheniformis* RF5-12 could produce non-ropy exopolysaccharides (Fig. S5).

*Ligilactobacillus salivarius* MP3 potentially inhibited the growth of *K. rhizophila* MIII and *C. acnes* subsp. *acnes* DSM 1897^T^. *K. rhizophila* can cause bovine mastitis^17^ and septicemia in immunodeficient patients, invasive surgery, and catheter placement. Nevertheless, several strains of *K. rhizophila* widely exhibited antimicrobial resistance^18^. *L. salivarius* was the potential tool for limiting pathogenic growth in this study. Guerrero Sanchez, et al.^19^ reported that *L. salivarius* could prevent enteropathogenic infection in piglets and calves. In addition, *L. salivarius* inhibited *Staphylococcus aureus* by secreting the LysM-containing peptidoglycan-binding protein and protein peptidase M23B^20^. Regarding the milk lipid depression of ruminants caused by *C. acnes*, no specific *L. salivarius* target has been reported for *C. acnes* in animals. Therefore, *L. salivarius* is a potential bacterium in terms of bactericidal activity, particularly against *K. rhizophila* and* C. acnes.*

In the present study, EPSs were produced from *S. luteliensis* RF1-5 and *B. licheniformis* RF1-12. The EPS production of *Bacillus licheniformis* RF5-12 was comparable to that reported in previous studies of Asgher, et al.^21^ and Petrova, et al.^22^. The antioxidant capacity and antimicrobial activity of *S. luteliensis* strain RF1-5 and *B. licheniformis* strain RF1-12 should be investigated in further research. According to these findings, the goat rumen is a possible source of antimicrobial compound-producing bacteria that could be used as alternatives in animal supplements and agricultural industries.

### The probiotic characterization of strain MP3

**Figure 3:**
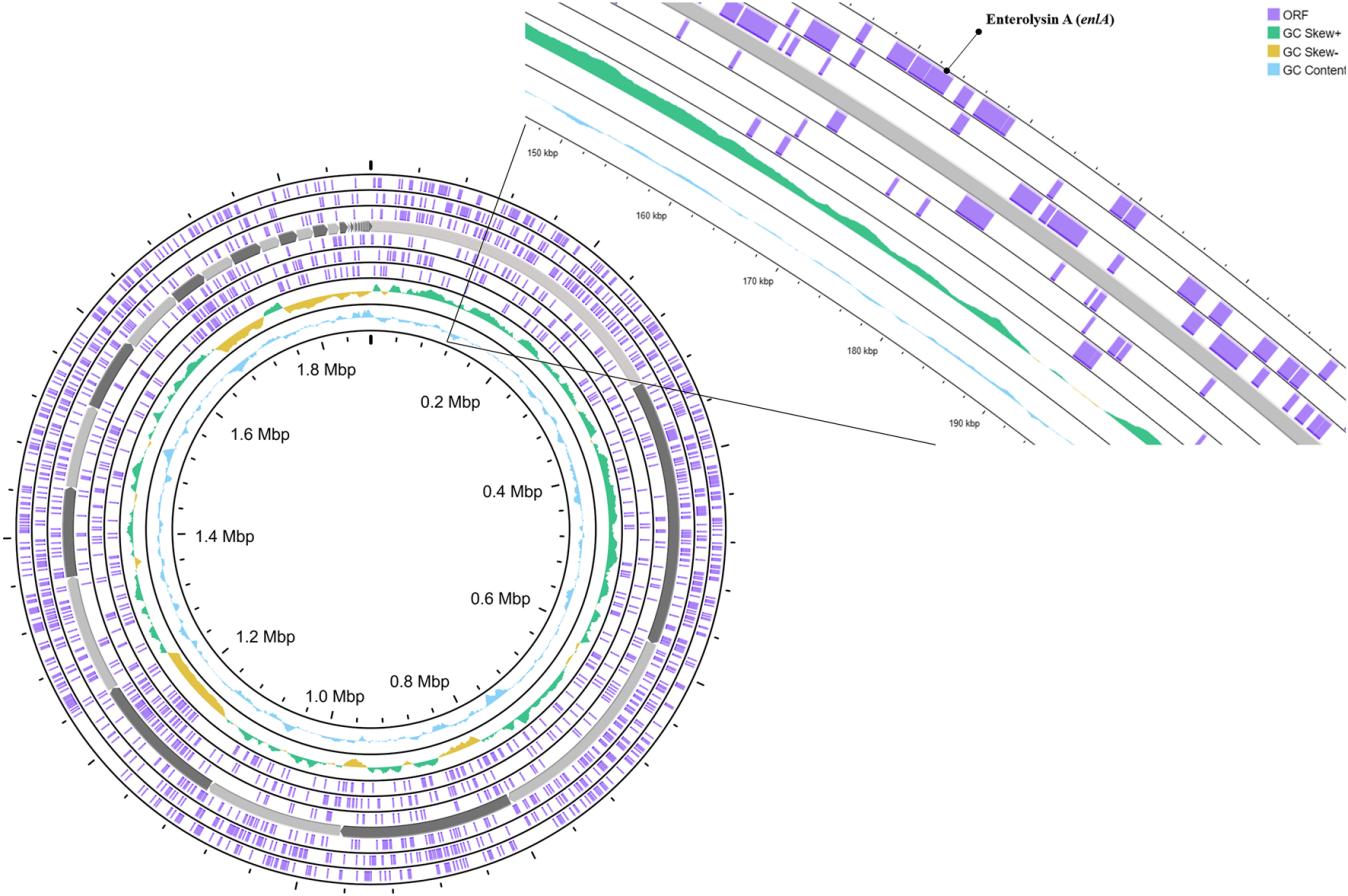
Circular genomic map of *Ligilactobacillus salivarius* MP3 visualized using a CG viewer. The open reading frame (ORF) is shown. The inner circle shows the genome size (1,876,541 bp).

The complete genome of *Ligilactobacillus salivarius* MP3 encompassed 1,876,541 bp with a GC content of 32.6% (Fig. 3). Microorganisms with lower GC contents were typically characterized by reduced energy consumption and metabolic rates, resulting in enhanced genome stability. Strain MP3 displayed the highest average nucleotide identity (ANI) values compared to *L. salivarius* DSM 20555^T^, with ANIb and ANIm values of 96.9% and 97.3%, respectively. These findings strongly support the classification of strain MP3 as a member of *L. salivarius* species. The growth pattern of *L. salivarius* MP3 is illustrated in Fig. S6. Strain MP3 entered the log phase approximately 3 h after incubation, maintaining stable bacterial cell growth for up to 15 h post-incubation, with the pH stabilizing at 4.75. Strain MP3 survived at pH 2, pH 3, 0.3% bile salt, and 1.0% bile salt (Table S5). The survival rates from gastrointestinal conditions, including pH 2, pH 3, 0.3% bile, and 1.0% bile, were 56.00%, 78.59%, 90.63%, and 86.73%, respectively. Additionally, several genes in strain MP3 supported the resistance in acid and bile conditions, as shown in Table S6. Furthermore, strain MP3 demonstrated the ability to thrive under extreme pH conditions. In addition to acid and bile tolerance, the expression of genes associated with probiotic attributes was also altered in strain MP3. Notably, it carried the *bsh* gene, which is known for its involvement in cholesterol-lowering activity. Furthermore, the genome of strain MP3 harbored putative genes, including *clpB, IspA,* and *tuf*, which play vital roles in immune system modulation. The critical regulatory genes involved in bile salt tolerance in *L. salivarius*, including beta-N-acetylhexosaminidase, PTS mannose transporter subunit IIB, and type 1 glutamine amidotransferase, play crucial roles in peptidoglycan synthesis and the PTS system^23^.

**Figure 4:**
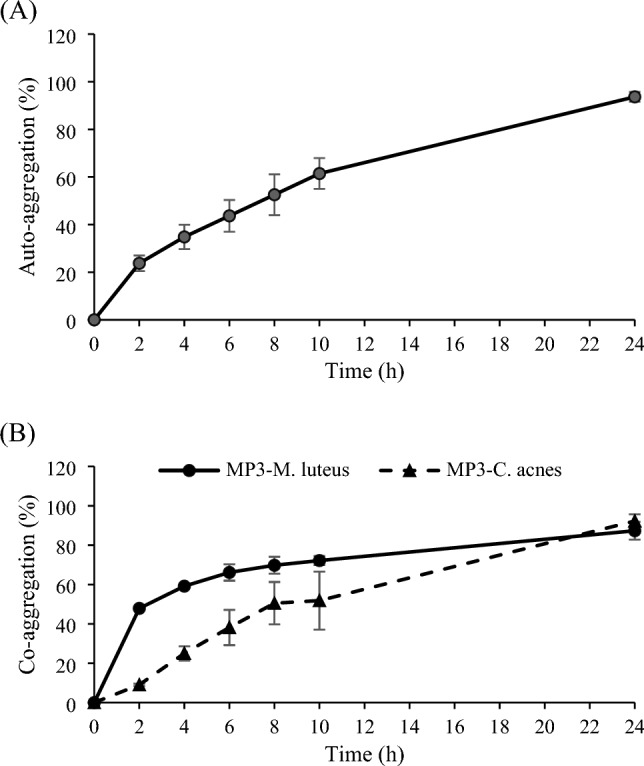
The percentage of auto-aggregation (**A**) and co-aggregation (**B**) with *Cutibacterium acnes* subsp. *acnes* DSM 1897^ T^ (black triangle) and *K. rhizophila* MIII (black circle) by strain MP3. The data are presented as the means ± standard deviations.

MP3 exhibited 23.0 ± 0.6% cell surface hydrophobicity. The auto-aggregation ability of strain MP3 was 93.6 ± 0.2% (Fig. 4A). In addition, the co-aggregation abilities of strain MP3 with *K. rhizophila* MIII and of strain MP3 with *C. acnes* subsp. *acnes* were 87.3 ± 4.5% and 92.2 ± 3.4%, respectively (Fig. 4B). Furthermore, the potential adhesion of strain MP3 to Caco-2 cells was 76.1 ± 2.2%. Adhesion to the host was supported by representative genes such as *srtA, dltA, Mub, groS,* and *glnA* (Table S6).

The impressive adhesion of strain MP3 was supported by class A sortase (*srtA*), the D-alanyl-lipoteichoic acid biosynthesis protein *dltD,* and D-alanylation of LTA (*dltA)*^24^. The ability of strain MP3 to auto-aggregate was greater than that of *L. salivalius* VKPM B-2214 (56.0 ± 1.1%)^25^, *L. salivarius* P851b1 (57.8 ± 1.3%), and *L. salivarius* P1041b1 (66.0 ± 2.6%)^26^. Variation in hydrophobicity was found for some *Lactobacillus* isolates (20.09–70.20%) in the study of Ait Seddik, et al.^26^. Strain MP3 exhibited better adhesion activity than LGG with different adhesion genes. The LGG strain lacked the *glnA* gene, which was a moonlighting protein, unlike the MP3 strain. Moonlighting proteins frequently adhere to host tissues. These proteins included glutamine synthetase (*glnA*), glucose-6-phosphate isomerase (*gpi*), enolase (*eno*), and glyceraldehyde-3-phosphate dehydrogenase (*gap*). Therefore, strain MP3 had potential adhesion to host tissue more than LGG.

The bacterial strains MP3 and LGG exhibited a hazy to greenish area (α-hemolysis) around their growth on sheep blood agar, indicating their lack of hemolytic activity. Hemolysis is an acknowledged attribute of pathogenic microorganisms that enhances their capacity to induce disease. On the other hand, it is essential to consider the lack of hemolytic activity while selecting probiotics^27^. For the genomic risk assessment, the pathogenicity of *L. salivarius* MP3 compared to that of *L. rhamnosus* GG and *L. salivarius* CGMCC20700 was presented in Table S7. The results demonstrated that MP3, LGG, and LS were non-human pathogens. The antibiotic resistance genes (ARGs) in strain MP3 were *VanT, tet(M), fexB,* and *ErmC,* similar to those in the probiotic strain *L. salivarius* CGMCC20700, in which the *Van*T gene was found*.* The antibiotic susceptibility of strain MP3 displayed resistance to amoxicillin, ceftazidime, clindamycin, erythromycin, kanamycin, tetracycline, oxacillin, bacitracin, trimethoprim, amoxiclav, vancomycin and carbenicillin. On the other hand, the sensitivity of the MP3 strain to antibiotic disc appeared in ampicillin, cefotaxime, ciprofloxacin, gentamicin, streptomycin, imipenem, neomycin, and rifampicin.

The ARGs of strain MP3 correlated with the antimicrobial susceptibility phenotype. The genes associated with increased resistance to clindamycin, erythromycin, and tetracycline were *ermC and tet(M).* Similarly, *L. salivarius* CGMCC20700 was reported tetracycline (*tetM, tetL*) and macrolide (*ermC*) resistance-related genes^28^. Tetracycline and erythromycin are commonly used for livestock treatment. Mobile genetic elements are frequently linked to genetic determinants that contribute to resistance to these antibiotics^29^. Furthermore, strain MP3 displayed a resistance phenotype to trimethoprim and vancomycin. Previous research has shown that *Lactobacillus* strains demonstrated high intrinsic resistance to trimethoprim and vancomycin^29^. Conversely, another study of *L. salivarius* revealed that this bacterium was susceptible to ampicillin, gentamicin, streptomycin, erythromycin, clindamycin, tetracycline, and chloramphenicol. However, this bacterium also exhibited kanamycin and vancomycin resistance. Even if the bovine-derived probiotic *L. salivarius* strain consisted of antibiotic resistance genes, the study indicated a low potential for horizontal transfer to cows^30^.

Furthermore, KEGG annotation revealed 21 different categories of genes. The most closely related terms were involved in carbohydrate metabolism (17.8%), genetic information processing (16.1%), protein families: genetic information processing (13.3%), nucleotide metabolism (7.9%), protein families: signaling and cellular processes (6.8%), amino acid metabolism (5.8%), metabolism of cofactors and vitamin (4.4%), and lipid metabolism (3.9%). The functional annotation revealed that the main products were lactate, acetate, propionate, and butyrate. According to the dbCAN analysis, the carbohydrate-related enzymes were annotated as 22 glycoside hydrolases (GHs), 23 glycosyltransferases (GTs), 3 carbohydrate esterases (CEs) and 3 carbohydrate-binding modules (CBMs). The GTs involved in carbohydrate metabolism participate in carbohydrate biosynthesis (i.e., oligosaccharides and polysaccharides). In addition, GH enzymes were considered to hydrolyze complex carbohydrates^31^. These enzymes enhanced the ability of strain MP3 to potentially utilize cellulose and hemicellulose.

**Figure 5:**
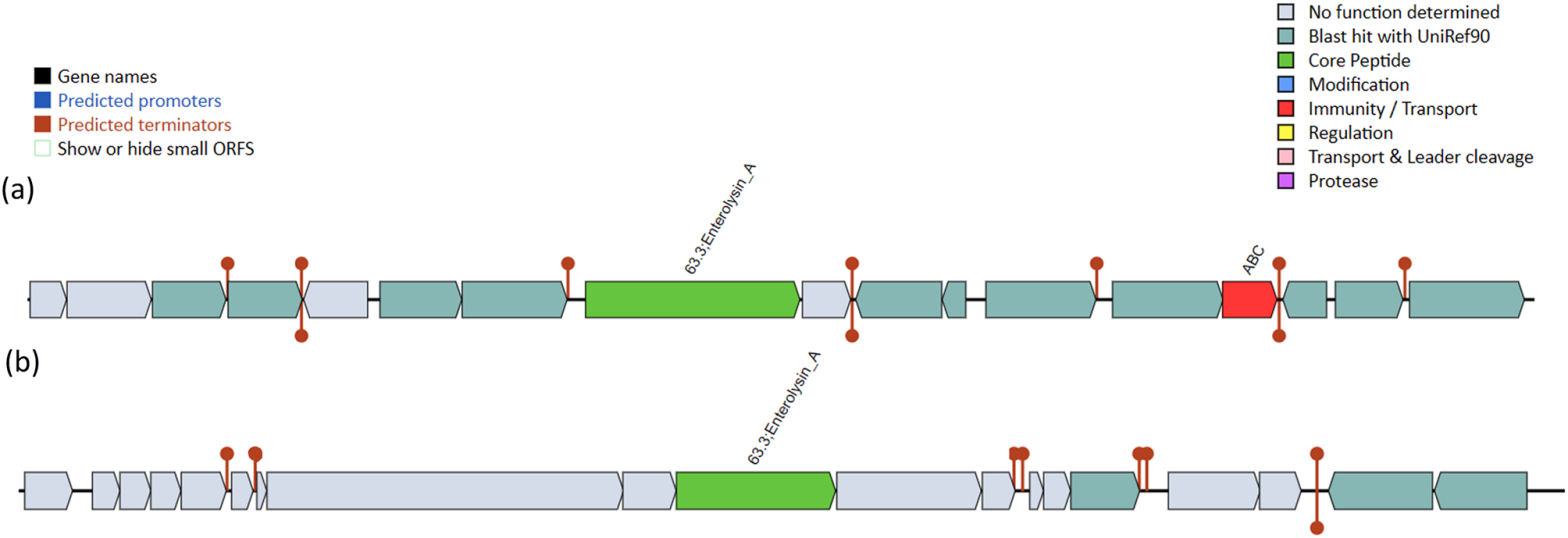
The bacteriocin clusters of strain MP3.

According to the BAGEL4 analysis, strain MP3 contained enterolysin A at contig 3.3 (starting at 172,580 and ending at 193,087) (Fig. 3). Contig 3.3 contained enterolysin A as a core protein, one ABC Arginine transport ATP-binding protein, and several open reading frames (ORFs) (Fig. 5). Enterolysin A was a class III bacteriocin that was first discovered in *Enterococcus faecalis* LMG 2333. Enterolysin A was found in *L. salivarius, E. hirae*^32^, *L. fermentum,* and *L. paracasei*^33^*.* Additionally, enterolysin A suppressed the growth of *Fusobacterium nucleatum*and *Clostridium difficile*^34^*.* Therefore, the antimicrobial activity of strain MP3 was demonstrated through the addition of lactic acid, acetic acid, hydrogen peroxide, and bacteriocin to *K. rhizophila* and *C. acnes* subsp. *acnes*.

The antimicrobial activity of strain MP3 showed its potential to antagonize pathogenic bacteria, including *L. monocytogenes* ATCC 19115, *S. aureus* ATCC 25923, *E. coli* ATCC 25922, *B. cereus* ATCC 6633 and *K. rhizophila* MIII (Fig. S7). Raw milk can be contaminated with *L. monocytogenes, E. coli*, and *B. cereus*, which can cause foodborne illnesses in humans and illness in dairy cattle^35–37^. The inhibitory effects of *L. salivarius* on *S. aureus*, *E. coli*, and *L. monocytogenes* were similar to those described in previous studies^38–40^. The utilization of MP3 could be an effective method for treating listeriosis and gastrointestinal illness by alleviating the potential negative impacts of subclinical carriage of *L. monocytogenes*, *E. coli*, and *B. cereus* on the gut health of animals. The production, induction, and characterization of enterolysin A from MP3 should be further investigated.

In conclusion, *L. salivarius* MP3 represented a bacteriogenic strain supported by the ability to adhere to host cells, tolerate GIT conditions, and produce antimicrobial substances. The antagonistic effect of strain MP3 on the milk fat depression caused by *C. acnes* was reported in this study. As a specific host probiotic, strain MP3 should be further evaluated for the risk of lateral antibiotic resistance gene transfer in vitro before supplementation of dairy ruminants.

## Methods

### Animal sample collection and lactic acid bacterial isolation

The animal experiment was evaluated by the ethical authority, received approval from the Chulalongkorn University Animal Care and Use Committee, Thailand (CUACUC; Approval No. 2231044) and was conducted in accordance with the relevant regulations of the authorities and the ARRIVE guidelines (https://www.arriveguidelines.org).

The six rumen fluid samples were obtained from donor lactating goats (Saanen × Thai native). The donors received a total mixed ration diet of 40:60 (roughage: concentrate) using corn silage as a roughage source. The rumen fluid samples were provided by the Veterinary Student-training Center, Department of Animal Husbandry, Faculty of Veterinary Science, Chulalongkorn University, Nakhon Pathom. The rumen fluid samples were collected at 4 h post-feeding. The samples were transferred to the laboratory in an anaerobic container by using AnaeroPack® (MITSUBISHI Gas Chemical America, Inc.). The rumen samples were prepared for bacterial isolation in an anaerobic chamber (Bactron, Sheldon Manufacturing, Inc.). After that, the rumen samples were passed through a 3-layer cheesecloth to remove the feed particles. All the rumen samples were tenfold serially diluted in phosphate buffer solution. The dilutions at 10^–4^ to 10^–8^ were spread on De Man Rogosa and Sharpe (MRS; Difco®) agar, peptone yeast extract glucose (PYG) agar, and modified peptone yeast extract fructose (mPYF) agar^41^ and incubated in an anoxic atmosphere (N_2_ 90%: H_2_ 5%: CO_2_ 5%) at 39 °C for 48 h. The colony was restreaked on MRS agar supplemented with 0.3% (w/v) CaCO_3_ for screening of lactic acid bacteria and incubated for 48 h at 39 °C. The lactic acid bacterial isolates were maintained in MRS media. All colonies that exhibited a clear zone were picked and stored at -20 °C in 15% (v/v) glycerol stock.

### Identification methods

Phenotypic characterization was performed for all the LAB strains. The cell morphology, colony appearance, and culture characteristics of the LAB strains were determined on MRS agar after incubating for 48 h. The physiological and biochemical characteristics were evaluated at different temperatures (15 °C, 30 °C, and 45 °C), pH (3.0, 6.0 and 9.0), and NaCl concentrations (3%, 6%, and 8%). The acid production from L-arabinose, D-cellobiose, D-fructose, D-galactose, D-lactose, D-maltose, D-mannitol, D-mannose, D-melibiose, D-raffinose, L-rhamnose, D-ribose, salicin, D-sorbitol, D-sucrose, D-trehalose and D-xylose was determined according to previous methods Tanasupawat, et al.^42^. Additionally, nitrate reduction, catalase, gas generation (CO_2_), and arginine dihydrolase test were determined. All the phenotypes were analyzed in the hierarchical cluster using SPSS v29. The 16S rRNA gene of the LAB strains was prepared and purified following the protocol of the AccuPrep Genomic DNA Extraction Kit (Bioneer Pacific, Australia). DNA sequencing was carried out at Macrogen, Inc. (Korea) with universal primers (27F, 1492R). The sequence similarity values related to the strains were compared on the EzBioCloud server^43^. The phylogenetic tree was constructed using the neighbor-joining (NJ) method via MEGA X^44^.

For novel species, phenotypic characteristics were evaluated. After 48 h of culture on PYG agar, the cell morphology, colony appearance, and colony pigmentation were investigated. Cell morphology was observed under a scanning electron microscope (JEOL, Japan) after cultivation at 39 °C for 48 h. The flagella were stained following the method of Forbes^45^ after incubation at 37 °C for 18 h. The ability to grow at different temperatures (20, 22, 30. 37, 39, 45, and 48 °C), pH (4.0–10 with 0.5 interval scale), and NaCl concentration (1–10% w/v) were determined in PYG broth. Acid production from carbohydrates was evaluated by peptone yeast extract medium supplemented with 1% (w/v) substrate. The carbohydrate substrates in this experiment consisted of amygdalin, D-adonitol, L-arabinose, D-arabitol, L-arabitol, D-cellobiose, dulcitol, esculin ferric citrate, erythritol, fructose, D-galactose, glycerol, inositol, inulin, D-lactose, D-maltose, D-mannose, D-melezitose, D-melibiose, D-raffinose, L-rhamnose, D-ribose, D-saccharose, salicin, D-sorbitol, L-sorbose, starch, D-tagatose, D-trehalose, xylitol, and D-xylose. The physiological characteristics of mPRGC5^T^ were observed the starch hydrolysis, casein hydrolysis, nitrate reduction, arginine hydrolysis, gelatin liquefaction, indole production, methyl red, and Voges-Proskauer test and hydrogen sulfide production. The enzymatic activities were determined by using an API® ZYM kit (bioMérieux SA). Chemotaxonomy was analyzed by using freeze-dried cells cultivated under optimal growth conditions. The fatty acid profile was extracted and detected by gas chromatography according to Sasser^46^. Polar lipids were extracted from freeze-dried cells and identified by two-dimensional TLC following the protocol of Minnikin, et al.^47^. For novel taxa, the concentrations of acetate, propionate, butyrate, valerate, *iso*-butyrate, and *iso*-valerate were determined using gas chromatography (GC-FID).

The genomic DNA of the 16S rRNA gene was extracted and sequenced. The contig DNA sequence was generated by using BioEdit software version 7.2.5. The contig sequences of the closely related strains were compared with those of the EzBioclound server. The evolutionary relationships among the bacterial strains were represented by phylogenetic trees. The construction of phylogenetic trees was based on the neighbor-joining, maximum-likelihood, and maximum-parsimony algorithms using the MEGA X program. The program was set to calculate by the Kimura-2-parameter model with 1000 replications. Thresholds of 98.7%, 94.5%, and 86.5% 16S rRNA gene sequence identity were indicated for novel species, genera, and families, respectively^48^. The assembled draft genomes of MP3 and mPRGC5 were deposited in the GenBank database under accession numbers JANFQI000000000 and VTOY00000000, respectively. The overall genome related index (OGRI) was calculated as the average nucleotide identity (ANI) value, the digital DNA‒DNA hybridization (dDDH) value, and the average amino acid identity (AAI) score to evaluate the species distinct from the nearest type strains. ANI and dDDH values were analyzed using the JSpecies WS web-based tool^49^ and the Genome-to-Genome Distance Calculator (GGDC 3.0) with the BLAST + method of formula 2 for the draft genome^50^. The cutoff values of ANI and dDDH, considered to indicate similar species, are > 95% and 70%, respectively^51^. The AAI value was calculated via a web-based service^52^. The genome phylogenetic tree was reconstructed by the type (strain) genome server^50^. The functional genes were annotated using rapid annotation using subsystem technology (RAST)^53^, the DDBJ fast annotation and submission tool (DFAST)^54^, and the KBase application^55^. The metabolic pathways were analyzed by the Kyoto Encyclopedia of Genes and Genomes (KEGG) with BlastKOALA^56^. Moreover, the circular genome was visualized and explored using Proksee^57^.

### Volatile fatty acid and exopolysaccharide production

The supernatants of all the strains inoculated in MRS broth were collected and stored at −20 °C for volatile fatty acid analysis. Gas chromatography with flame-ionization detection (Agilent 7890B) was applied to evaluate the concentrations of volatile fatty acids, including acetate, propionate, butyrate, and valerate. The carrier gas was helium, and the flow rate was 1.0 mL.min^−1^. In addition, the flows of hydrogen gas and air zero were set at 30 and 400 mL.min^−1^. At the inlet, the sample was split 10:1, and the injection volume was 1 µL. The oven temperature was programmed as described by Darwin, et al.^58^. Standard calibration was performed using a volatile fatty acid mixture analytical standard (Merck®, Germany).

Glucose in the MRS agar medium was replaced with sucrose (2%) for exopolysaccharide (EPS) screening^59^. The LAB strains were streaked on EPS agar and incubated at 37 °C under anaerobic conditions for 48 h. The EPS-producing strain exhibited a ropy phenotype, as detected by touching the colony with an inoculated loop. The colonies showed an unbreakable strand or presented a mucoid non-ropy phenotype (slime formation, glistening, smooth colony) on the plate.

### Spot resistant study for antimicrobial activity

The protocol followed the method of Luo, et al.^60^. The 24 h inoculum of bacterial strains in MRS broth was used in this test. Anaerobes were incubated at 37 °C in an anaerobic chamber. All the isolated strains were centrifuged at 7000 × g at 4 °C for 10 min to harvest the cell pellet, after which the cells were resuspended in phosphate buffer solution to adjust the OD600 to 1.0. For screening antimicrobial activity, the target strain was *Cutibacterium acnes* subsp. *acnes* DSM 1897^ T^, which was the milk fat depression bacterium found in dairy ruminants. *C. acnes* was cultured in PYG broth for 72 h at 37 °C under anaerobic conditions, after which the bacterial cells were collected as described above. The antimicrobial activities of the strains against *Lactobacillus sakei* subsp. *sakei* JCM 1157^ T^*, E. coli* ATCC 25922^ T^*, Staphylococcus aureus* ATCC 25923^ T^*, Streptococcus agalactiae* 1611 and *K. rhizophila* MIII were screened*.* MRS medium was used for the preculture of *Lb. sakei* subsp. *sakei* JCM 1157^ T^ and brain heart infusion medium (BHI; Difco, Leeuwarden, The Netherlands) were used to grow the pathogens. Subsequently, *Lb. sakei* subsp. *sakei* JCM 1157^ T^ and the pathogen strains were incubated at 37 °C for 24, 48 and 72 h. First, 100 µl of *C. acnes* subsp. *acnes* DSM 1897^ T^*, Lb. sakei* subsp. *sakei* JCM 1157^ T^ and pathogens were swabbed on PYG, MRS, and BHI plates to make up the basal layer. Consequently, 2 µL of bacterial suspension was added to the center of the plate. All the strains were analyzed in triplicate and incubated at 37 °C for 24 h in the anaerobic chamber. The spot inhibition zone was measured as the diameter (mm) of the clear zone around the bacterial spot. All the strains that presented inhibitory activity were selected for further study.

### Evaluation of the probiotic properties of strain MP3

Strain MP3 was inoculated in MRS broth at 37 °C for 48 h under anaerobiosis. The samples were collected every 3 h to evaluate the cell concentration via a spectrophotometer at 600 nm, and the colony-forming units per milliliter (CFU/mL) were estimated through plate-serial dilution spotting. The growth curve was plotted to determine the exponential phase (log phase).

The acid and bile tolerance were determined following the modified method of Kingkaew, et al.^61^. The active culture of strain MP3 was inoculated into MRS broth adjusted to pH 2 and pH 3 or into MRS broth supplemented with 0.3% and 1.0% (w/v) bile salt. Subsequently, all the inoculates were incubated at 37 °C for 3 h. The viable cell counts were estimated by plate-serial dilution spotting, and all the plates were incubated at 37 °C for 24 h. The viable cell count was expressed as CFU/mL. The culture in normal MRS was used as control.

The cell surface hydrophobicity of strain MP3 was evaluated according to Cele, et al.^62^. This method was used to investigate hydrophobicity. The strain MP3 was cultured overnight in MRS broth at 37 °C in an anaerobic chamber. The cells were centrifuged (7,300 × g, 4 °C, 10 min) and washed in phosphate buffer solution (PBS). After that, the cell pellet was resuspended in 1.5 mL of PBS, and the optical density at 600 nm (OD_0_) was determined. The cell suspension was mixed with ethyl acetate at a ratio of 1:1. Then, the solution was vortexed for 2 min and incubated at room temperature for 1 h to separate the aqueous and organic phases. Finally, the aqueous phase sample was collected, and the optical density was measured at 600 nm (OD_1_). The hydrophobicity was calculated according to the following Eq. (1):

1 $$\% {\text{hydrophobicity}} = \frac{{{\text{OD}}_{0} {-}{\text{ OD}}_{{1}} }}{{{\text{OD}}_{0} }} \times 100\%$$

The auto-aggregation and co-aggregation assays were performed following the method of Li, et al.^63^. In brief, overnight culture of strain MP3 in MRS medium was used to collect the cells by centrifugation at 7,300 rpm for 10 min. The cells were washed 3 times with phosphate-buffered saline (PBS) and resuspended in 2 mL of PBS. The initial OD_600_ (A_0_) was evaluated. Subsequently, the bacterial solution was incubated at room temperature for 24 h. Afterwards, 100 µL of the upper part of the bacterial solution was taken at 2, 4, 6, 8, 10, and 24 h after standing to measure the optical density at OD_600_ (A_1_). The auto-aggregation rate was calculated according to Eq. 2. The bacterial solutions for co-aggregation were prepared as described above. The 2 mL of strain MP3 was mixed with 2 mL of the indicator strains (*C. acnes* subsp. *acnes* DSM 1897^ T^ and *K. rhizophila* MIII) and incubated at room temperature. The OD_600_ was measured after 0, 2, 4, 6, 8, 10, and 24 h of incubation. The percentage of co-aggregation was determined via Eq. 3.

2 $$\% {\text{auto-aggregation}}\frac{{{\text{A}}_{0} {-}{\text{ A}}_{{1}} }}{{{\text{A}}_{0} }} \times 100\%$$

3 $$\% {\text{co-aggregation}} = \frac{{{\text{A}}_{0} {-}{\text{ A}}_{{1}} }}{{{\text{A}}_{0} }} \times 100\%$$

An adhesion assay was performed to investigate the adhesion of strain MP3 to Caco-2 cells^61^. Caco-2 cells (5 × 10^5^ cells/mL) were used in this study. Cells of strain MP3 were prepared by overnight culture in MRS broth. The overnight culture was centrifuged at 7000× g and 4 °C for 7 min, after which the cell pellet was resuspended in fresh warm Dulbecco modified Eagle Minimum Essential Medium (DMEM) (N_0_). After the addition of the suspension of strain MP3 to the plate, three replicates were incubated at 37 °C in a 5% CO_2_ incubator for 90 min. Finally, the Caco-2 cells were washed with PBS and then lysed cells with 0.05% of Triton-X100 solution. The MP3 inoculum (N_0_) and adherent cells with Caco-2 cells (N_1_) were evaluated via the spot-plate technique. The percentage of adhesion capability was calculated using Eq. (4):

4 $${\text{Adhesion\,percentage }}\left( \% \right) = \frac{{{\text{N}}_{{1}} }}{{{\text{N}}_{{0}} }} \times 100$$

The hemolytic activity was measured to assess its safety. The MP3 and LGG strains were streaked onto blood agar plates containing 5% (v/v) sheep blood and incubated at 37 °C for 48 h. Hemolysis was detected in surrounding areas of bacterial growth. Beta (β) hemolysis refers to the presence of a clear zone surrounding bacterial growth, indicating positive activity. On the other hand, the presence of a greenish to brownish zone (α-hemolysis) or the absence of a zone (γ-hemolysis) were classified as non-hemolytic activity.

The antimicrobial sensitivity of strain MP3 was evaluated by the antimicrobial disk susceptibility test^64^. The antibiotic disks included ampicillin (10 mcg; AMP10), amoxiclav (30 mcg; AMC30), amoxycillin (10 mcg; AML10), carbenicillin (100 mcg; CAR100), ceftazidime (30 mcg; CAZ30), cefotaxime (30 mcg; CTX30), ciprofloxacin (5 mcg; CIP5), clindamycin (2 mcg; DA2), erythromycin (15 mcg; E15), gentamicin (10 mcg; CN10), kanamycin (30 mcg; K30), streptomycin (10 mcg; S10), tetracycline (30 mcg; TE30), oxacillin (1 mcg; OX1), bacitracin (0.04U; A), imipenem (10 mcg; IPM10), neomycin (30 mcg; N30), rifampicin (5 mcg; RD5), trimethoprim (25 mcg; SXT25) and vancomycin (30 mcg; VA30). All the strains were cultivated on Mueller–Hinton agar (MHA), after which the antimicrobial disks were added to the agar. The inoculated agars were incubated for 24 h at 37 °C in an anaerobic chamber. After incubation, antimicrobial resistance was indicated by the inhibition zone (diameter, mm) around the antibiotic disks. The grade of resistance was evaluated according to the criteria of the CLSI document M100-2A: resistant, mid-grade sensitive and sensitive.

Antimicrobial activity was evaluated using a spot-on-the-lawn assay^65^. The cell-free supernatant (CFS) was collected from 15 h of culture of strain MP3 by centrifugation at 10,000 × g for 10 min at 4 ℃. The CFS was sterilized by passing through a 0.22 µm filter membrane. The bottom layer of the plates on the lawn was composed of brain–heart infusion (BHI, HiMedia Laboratories, India) with 1.5% agar. This layer was overlain with a top layer of 0.7% soft agar mixed with 100 µL of active pathogenic culture. The pathogenic indicator strains consisted of *B. subtilis* JCM 1465^T^, *Kocuria rhizophila* MIII, *S. aureus* DMST 6512, Methicillin*-resistant S. aureus* DMST 20635, *E. coli* O157:H7, *E. coli* ATCC 3540. A volume of 10 µL of CFS was spotted on the indicator agar. The negative control consisted of 10 µL of MRS broth, while the positive control included the use of acetic acid as a reference. The plates were incubated at 37 °C for 24 h, after which the inhibitory zone was observed.

Probiogenomic analysis was performed via web-based tools following the protocol of Kingkaew et al.^66^. The draft genome of strain MP3 and two probiotic strains, *Lacticaseibacillus rhamnosus* GG ATCC 53103^T^ (accession no. FM179322, LGG) and *L. salivarius* CGMCC20700 (accession no. CP101685, LS), were annotated by the Rapid Annotation Server Technology server, DDBJ Fast Annotation and Submission Tool and the Pathosystems Resource Integration Center (PATRIC). The genome qualities were shown in Table S8. Pathogenicity was predicted using VirulenceFinder 2.0^67^, PathogenFinder 1.1^68^ and PlasmidFinder 2.1^69^. Antibiotic resistance genes were investigated via the Comprehensive Antibiotic Resistance Database (CARD), PATRIC and ResFinder 4.1. The dbCAN meta server^70^ was used to identify carbohydrate-active enzymes based on the CAZy database^71^. The bacteriocins, lactic acid, acetic acid and hydrogen peroxide were predicted from the genome data of strain MP3 to identify the specific antimicrobial product. The antimicrobial peptide gene clusters of strain MP3 were identified and visualized using BAGEL4^72^.

### Supplementary Information

Supplementary Information.

## Data Availability

The authors confirm that the DNA sequence data in this study have been deposited in the National Center for Biotechnology Information (NCBI) database (https://www.ncbi.nlm.nih.gov/). The data that support the findings of this study are available from the corresponding author upon reasonable request.
