# Supplementary material for: Anaerobic flora, Selenomonas ruminis sp. nov., and the bacteriocinogenic Ligilactobacillus salivarius strain MP3 from crossbred-lactating goats
Source: Sci Rep. 2024 Feb 28;14:4838. doi: 10.1038/s41598-024-54686-6 (PMC10901824; doi:10.1038/s41598-024-54686-6)
Supplement: Supplementary file 1 — Supplementary Information. [file 41598_2024_54686_MOESM1_ESM.docx]

Anaerobic flora, *Selenomonas ruminis* sp. nov., and the bacteriocinogenic *Ligilactobacillus salivarius* strain MP3 from crossbred-lactating goats

**Saranporn Poothong^1^, Somboon Tanasupawat^2*^, Somchai Chanpongsang^1^, Engkarat Kingkaew^3^, Chackrit Nuengjamnong^1,4**^**

^1^Department of Animal Husbandry, Faculty of Veterinary Science, Chulalongkorn University, Bangkok 10330, Thailand

^2^Department of Biochemistry and Microbiology, Faculty of Pharmaceutical Sciences, Chulalongkorn University, Bangkok 10330, Thailand

^3^Department of Biology, School of Sciences, King Mongkut’s Institute of Technology Ladkrabang, Bangkok 10520, Thailand

^4^Center of Excellence for Food and Water Risk Analysis (FAWRA), Faculty of Veterinary Science, Chulalongkorn University, Bangkok 10330, Thailand

*** Corresponding author:** Somboon Tanasupawat, Somboon.T@chula.ac.th

**** Corresponding author:** Chackrit Nuengjamnong, Chackrit.n@chula.ac.th


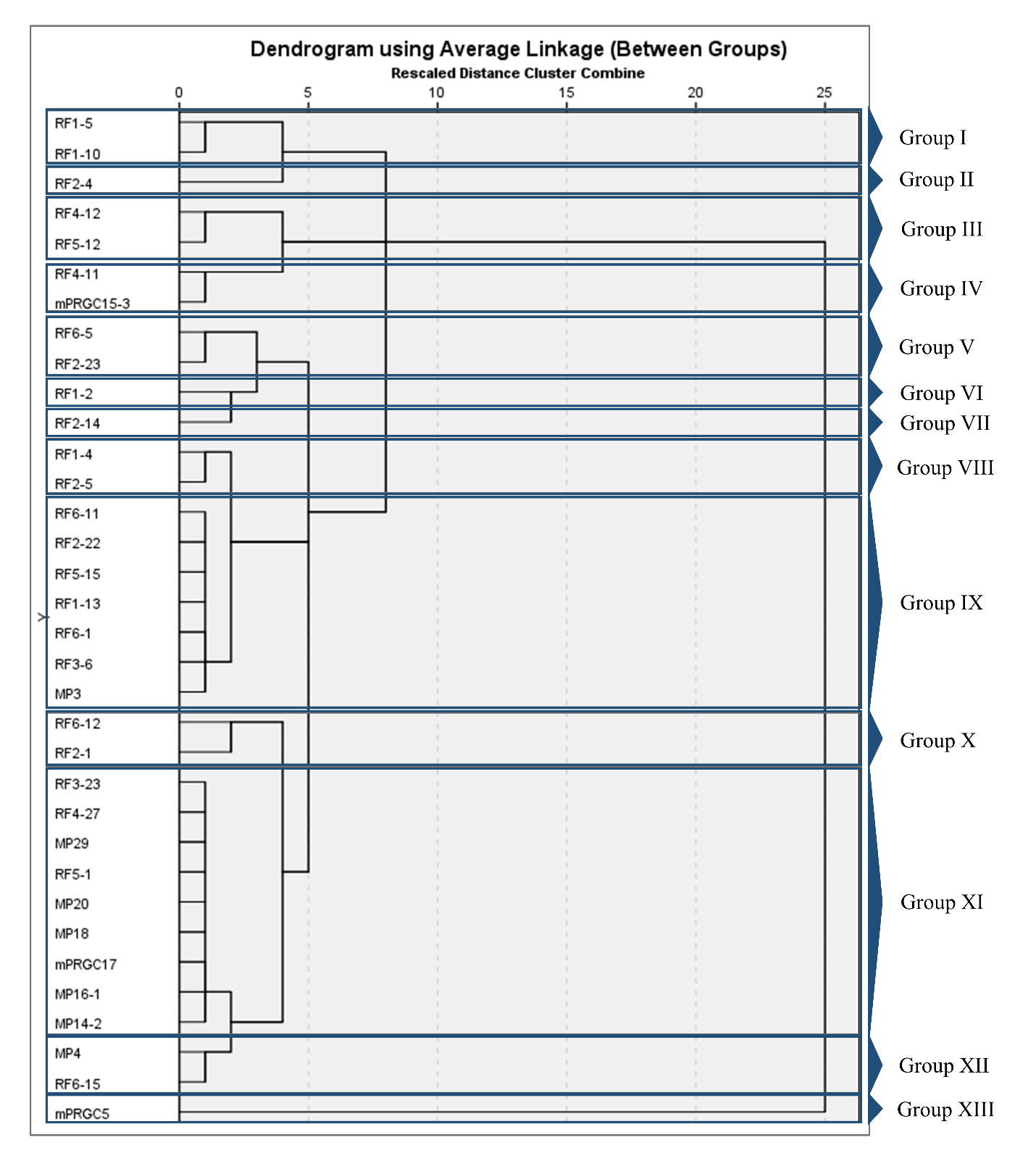


**Fig. S1**: Dendrogram of hierarchical cluster-based phenotypic characteristics.


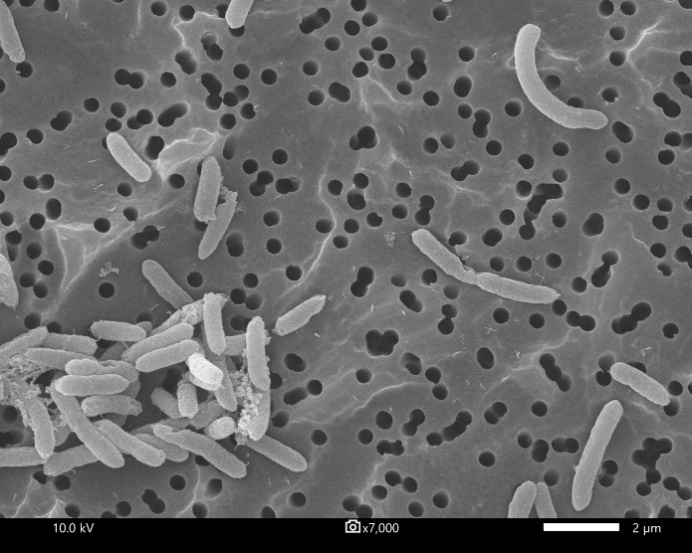


**Fig. S2:** Scanning electron micrograph of strain mPRGC5^T^ grew in PYG broth at 39 ℃ for 24 h.

**
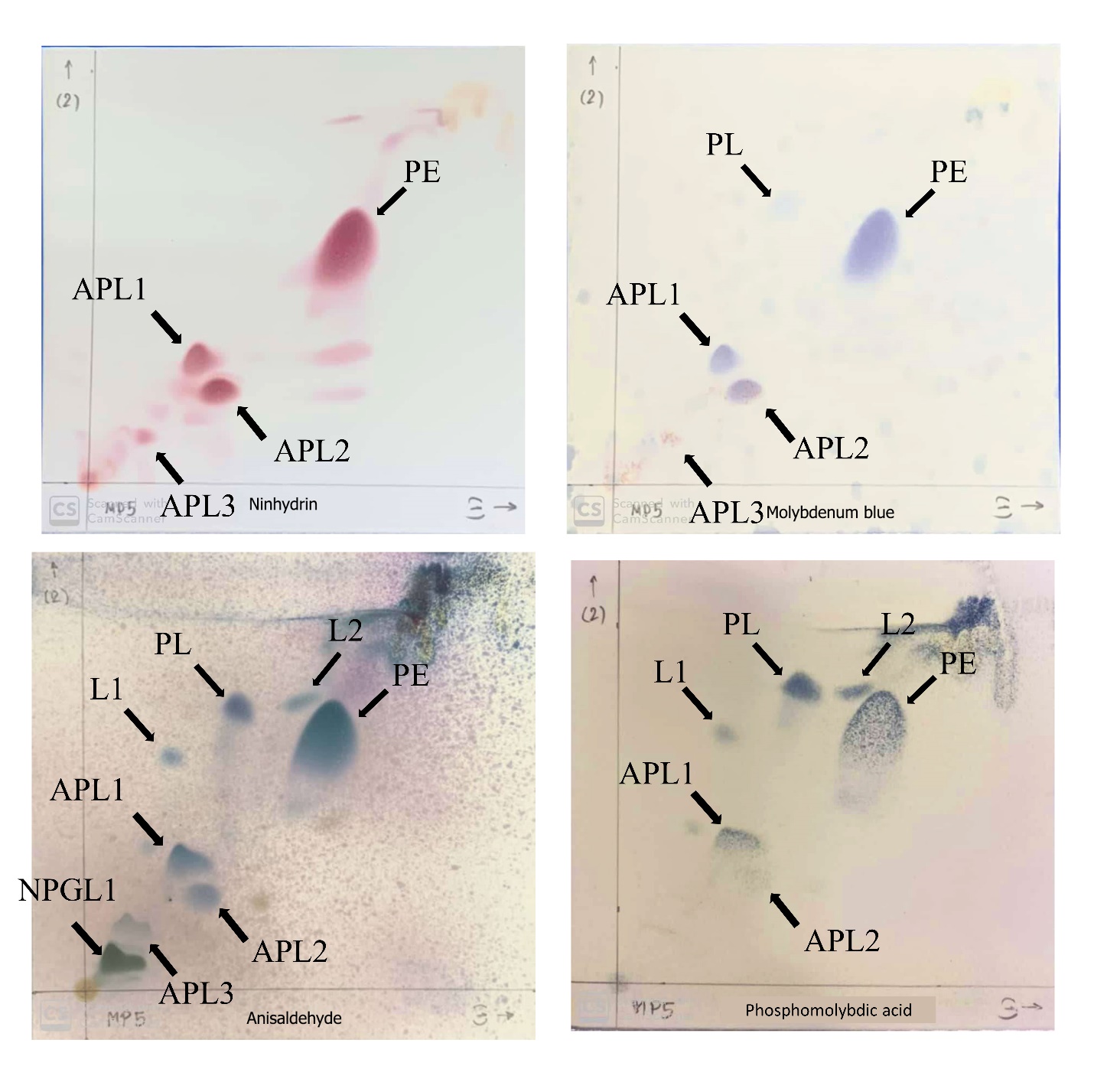
**

**Fig. S3:** Polar lipid profiles of strain mPRGC5^T^ based on two-dimensional thin layer chromatograms that were detected with ninhydrin, and Molybdenum blue, anisaldehyde, and phosphomolybdic acid as spraying reagent. PE, phosphatidylethanolamine; APL, aminophospholipids; NPGL, unidentified ninhydrin positive glycolipid; PL, phospholipid; L, unidentified lipids**.**


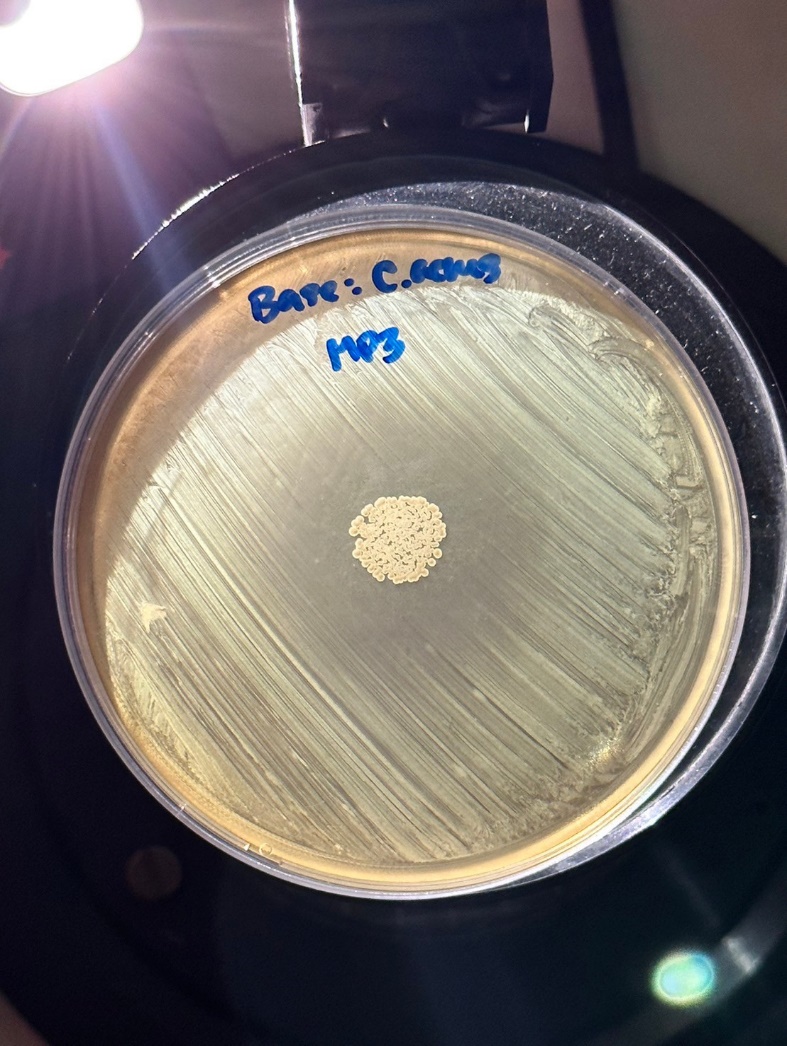

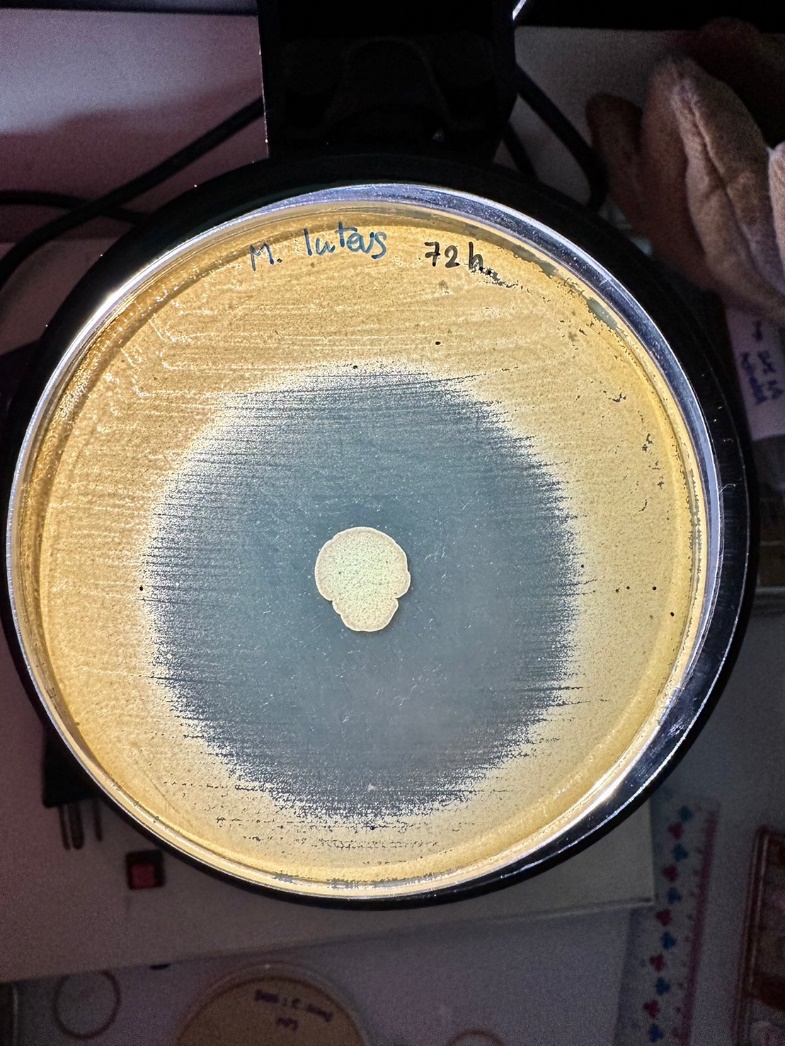


(B)

(A)

**Fig. S4:** The inhibitory activities of *Ligilactobacillus salivarius* MP3 against *Cutibacterium acnes* subsp. *acnes* (A) and *Kocuria rhizophila* (B) after incubated 37 °C for 72 h on brain heart infusion medium.


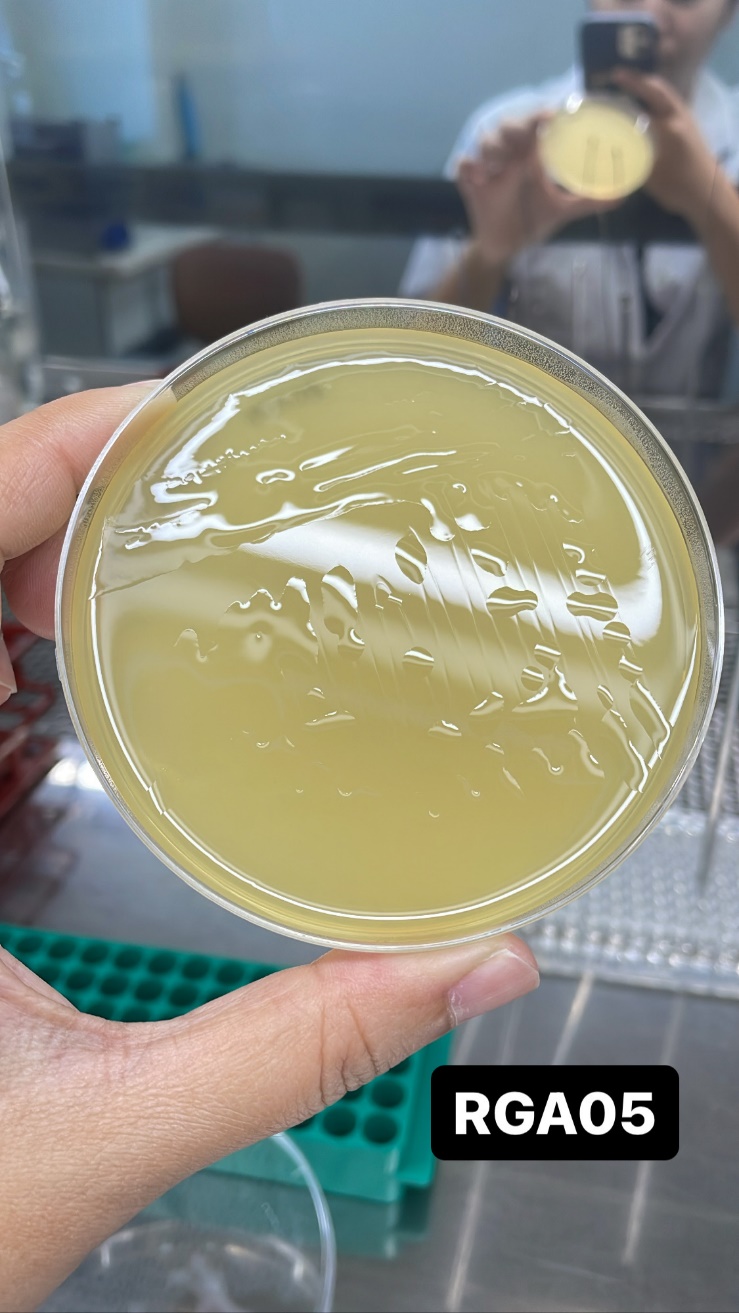

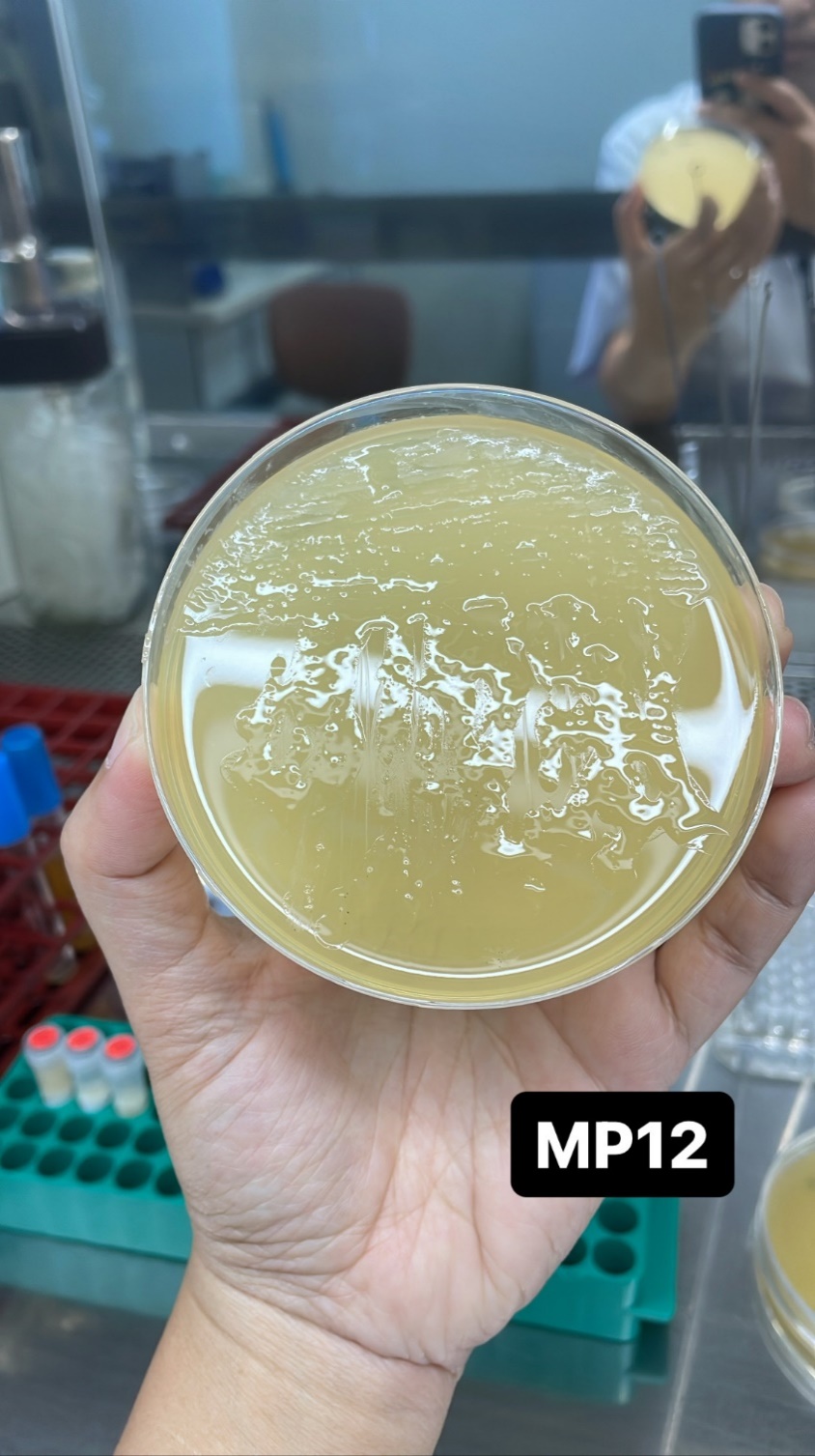


(B)

(A)

**Fig. S5:** Slime forming colonies of strain RF1-5 (A) and RF5-12 (B) on MRS agar supplemented with 2% sucrose.


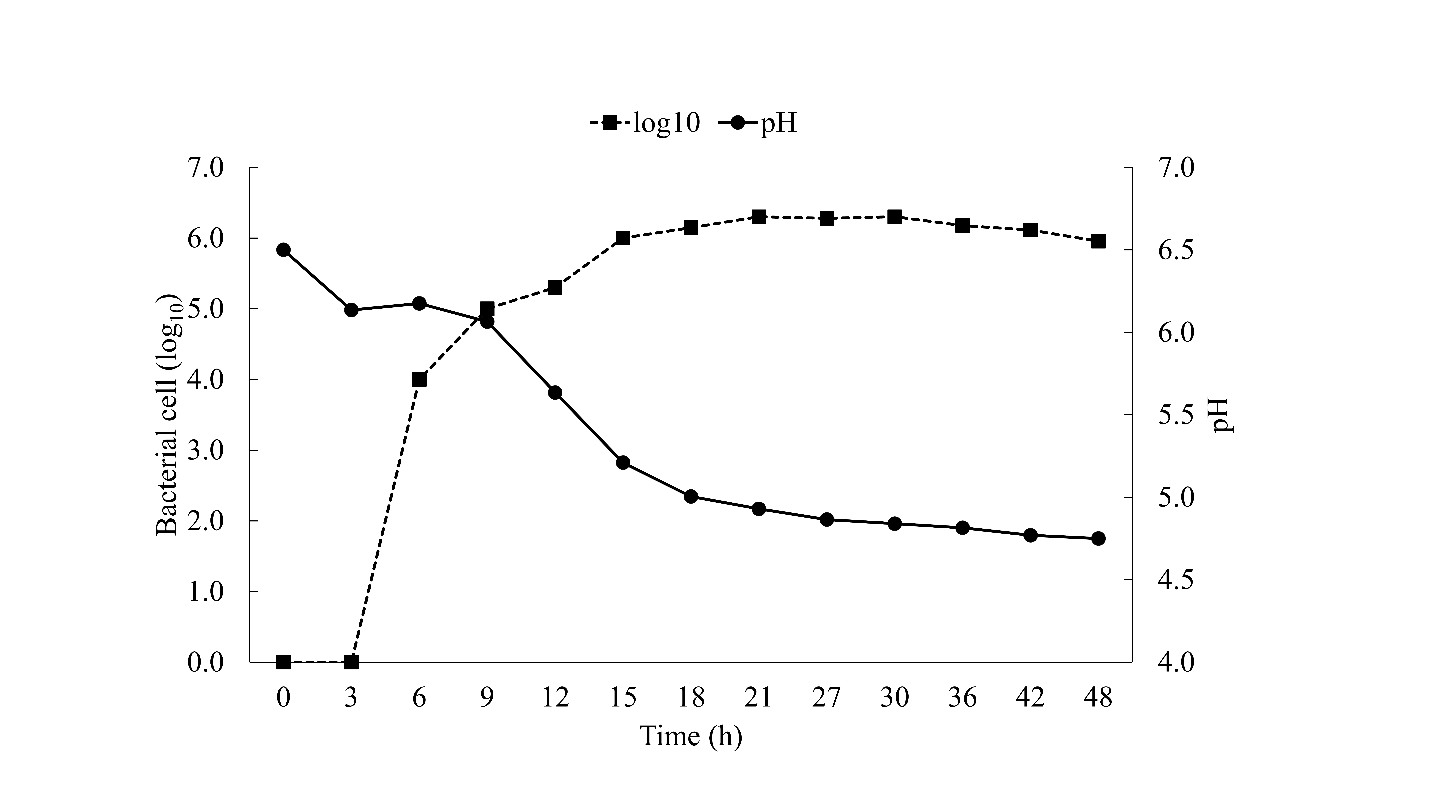


**Fig. S6:** The correlation of viable cell counts and pH along 48 h incubation at 37 ℃ of *L. salivarius* MP3.


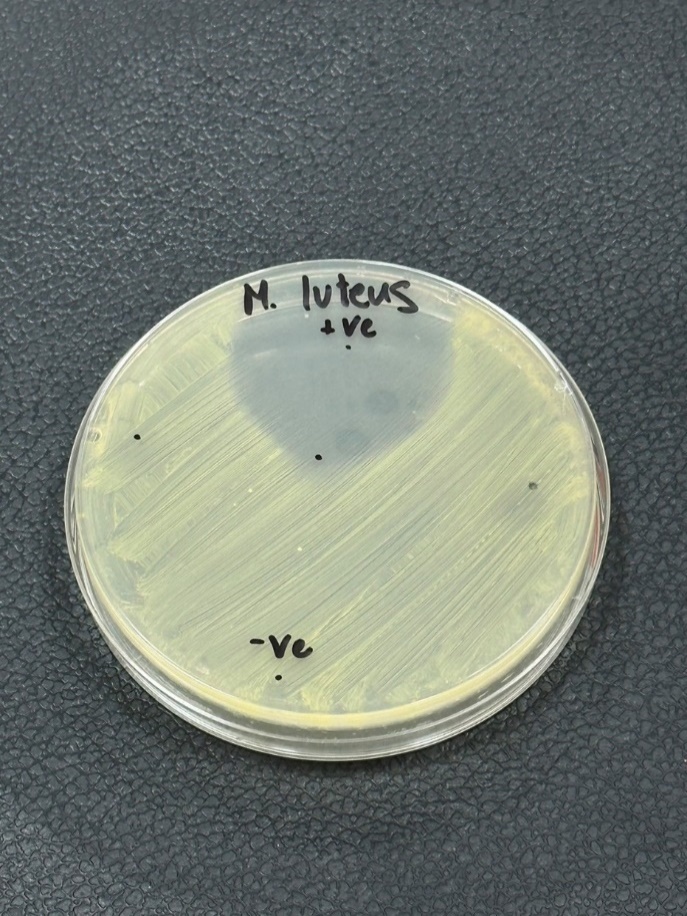

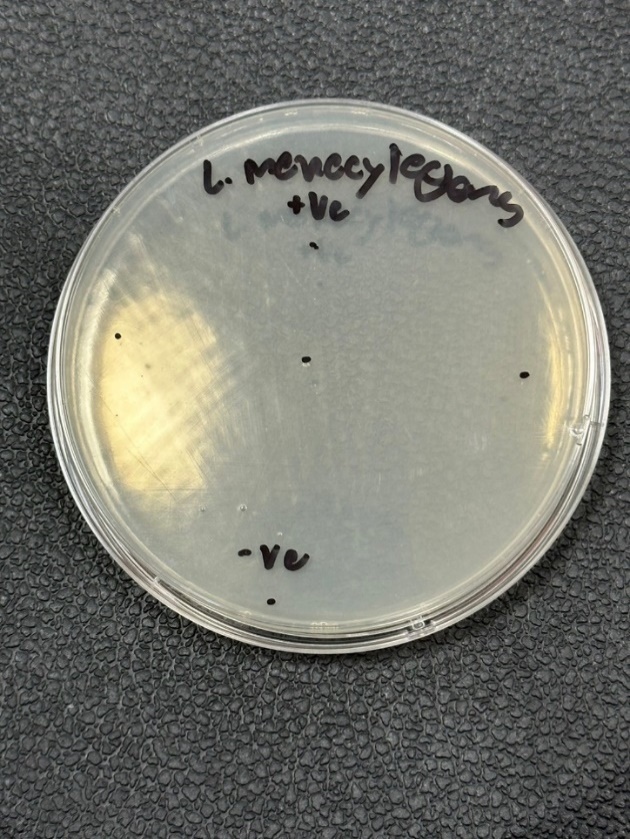

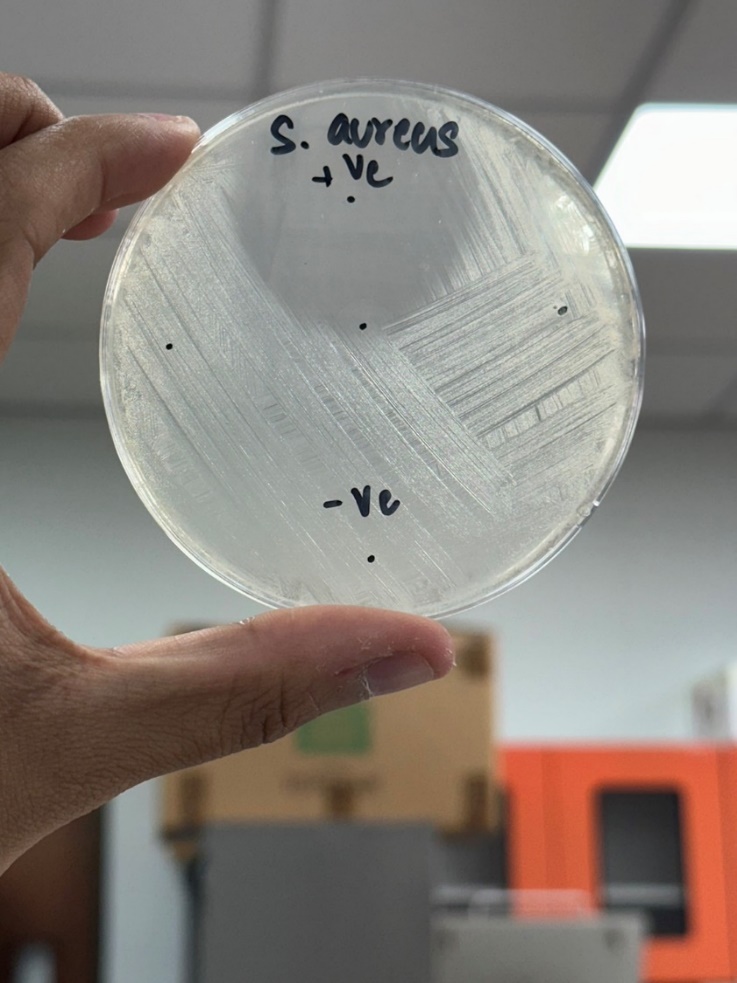

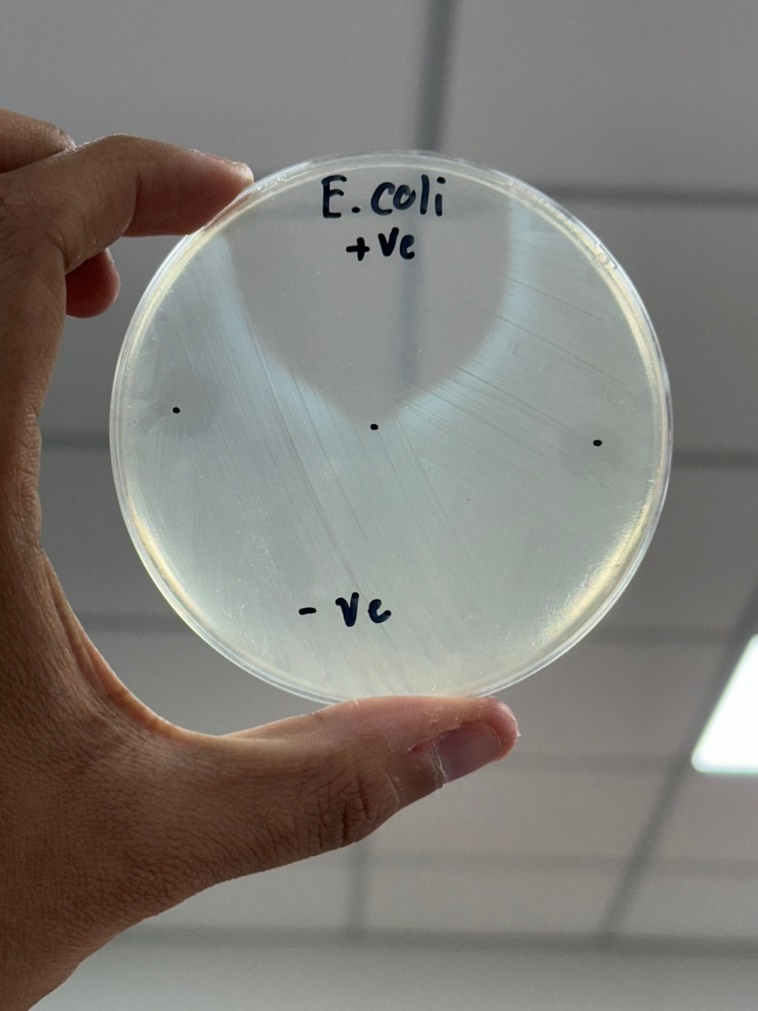

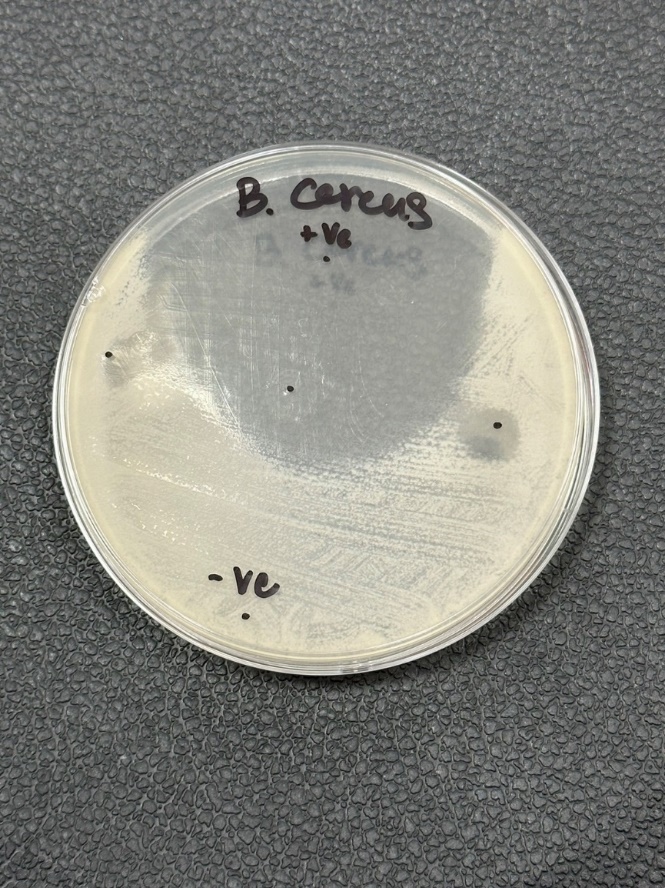


**Fig. S7:** The inhibitory effects of the CFS of strain MP3 to *L. monocytogenes* ATCC 19115, *S. aureus* ACC 25923, *E. coli* ATCC 25922, *B. cereus* ATCC 6633 and *M. luteus* MIII*.* The MRS broth and acetic acid were used as negative and positive control, respectively.

**Table S1:** Isolate number, isolation medium, nearest relatives, 16S rRNA gene sequence similarity of isolates.

| **Group** | **Isolate no.** | **Medium** | **Nearest relatives** | **Similarity (%)** | **Length (bp)** | **Accession no.** |
| --- | --- | --- | --- | --- | --- | --- |
| I | RF1-5 | MRS | *Streptococcus lutetiensis* CIP 106849^T^ | 99.01 | 1,330 | OR673714 |
|  | RF1-10 | MRS |  | ND | ND | - |
| II | RF2-4 | MRS | *Enterococcus gallinarum* NBRC 100675^T^ | 99.93 | 1,374 | OR673694 |
| III | RF5-12 | MRS | *Bacillus licheniformis* ATCC 14580^T^ | 99.70 | 1,364 | OR673692 |
|  | RF4-12 | mPYF |  | ND | ND | - |
| IV | RF4-11 | mPYF | *Mitsuokella jalaludinii* M 9^T^ | 99.64 | 1,399 | OR673702 |
|  | mPRGC15-3 | mPYF |  | 99.39 | 1,481 | OR673703 |
| V | RF6-5 | mPYF | *Limosilactobacillus oris* DSM 4864^T^ | 99.93 | 1,389 | OR673701 |
|  | RF2-23 | MRS |  | ND | ND | - |
| VI | RF1-2 | MRS | *Lacticaseibacillus rhamnosus* JCM 1136^T^ | 100.0 | 1,336 | OR673696 |
| VII | RF2-14 | PYG | *Lactobacillus amylovorus* DSM 20531^T^ | 99.85 | 1,378 | OR673695 |
| VIII | RF2-5 | PYG | *Enterococcus avium* ATCC 14025^T^ | 99.78 | 1,374 | OR673693 |
|  | RF1-4 | MRS |  | ND | ND | - |
| IX | MP3 | mPYF | *Ligilactobacillus salivarius* BCRC 14759^T^ | 99.80 | 1,562 | OR673697 |
|  | RF3-6 | mPYF |  | ND | ND | - |
|  | RF6-1 | mPYF |  | 99.79 | 1,445 | OR673698 |
|  | RF6-11 | MRS |  | ND | ND | - |
|  | RF2-22 | MRS |  | ND | ND | - |
|  | RF1-13 | MRS |  | 99.78 | 1,384 | OR673699 |
|  | RF5-15 | MRS |  | 99.79 | 1,410 | OR673700 |
| X | RF2-1 | MRS | *Pediococcus acidilactici* DSM20284^T^ | 99.50 | 1,416 | OR673710 |
|  | RF6-12 | MRS |  | 99.83 | 1,212 | OR673711 |
| XI | RF4-27 | PYG | *Parafannyhessea umbonata* DSM 22620^T^ | 99.92 | 1,329 | OR673704 |
|  | RF3-23 | PYG |  | ND | ND | - |
|  | MP14-2 | mPYF |  | 99.92 | 1,311 | OR673705 |
|  | MP16-1 | mPYF |  | ND | ND | - |
|  | mPRGC17 | mPYF |  | 100.00 | 1,257 | OR673706 |
|  | MP18 | mPYF |  | 100.00 | 1,230 | OR673707 |
|  | MP20 | mPYF |  | ND | ND | - |
|  | RF5-1 | mPYF |  | 100.00 | 1,221 | OR673708 |
|  | MP29 | mPYF |  | 100.00 | 1,256 | OR673709 |
| XII | MP4 | mPYF | *Sharpea azabuensis* JCM14210^T^ | 99.78 | 1,365 | OR670712 |
|  | RF6-15 | PYG |  | 99.78 | 1,366 | OR673713 |
| XIII | mPRGC5 | mPYF | *Selenomonas caprae* JCM 33724^T^ | 97.88 | 1,407 | LC487987 |

**Table S2:** Phenotypic characteristics of isolates.

| **Characteristics** | **I** | **II** | **III** | **IV** | **V** | **VI** | **VII** | **VIII** | **IX** | **X** | **XI** | **XII** | **XIII** |
| --- | --- | --- | --- | --- | --- | --- | --- | --- | --- | --- | --- | --- | --- |
| No. of isolate | 2 | 1 | 2 | 2 | 2 | 1 | 1 | 2 | 7 | 2 | 9 | 2 | 1 |
| Cell shape | Cocci | Cocci | Rods | Rods | Rods | Rods | Rods | Cocci | Rods | Tetracocci | Rods | Rods | Rods |
| Gram stain | + | + | + | - | + | + | + | + | + | + | + | + | - |
| Catalase | - | - | + | + | - | - | - | - | - | - | - | - | - |
| Gas from glucose | - | - | - | - | - | - | - | - | - | - | - | + | - |
| Growth at pH 3 | - | + | + | + | + | + | + | - | + | + | + | + | - |
| Growth at pH 9 | + | - | + | + | + | + | - | + | + | + | + | + | + |
| Growth at 15 °C | - | + | + | - | - | + | - | + | - | + | - | + | - |
| Growth at 45 °C | + | + | + | + | + | + | + | + | + | + | + | + | - |
| Growth in 3% NaCl | + | + | + | + | + | + | + | + | + | + | + | + | - |
| Arginine hydrolysis | + | + | + | + | - | - | - | - | - | + | + | - | - |
| Nitrate reduction | + | - | + | + | + | + | - | - | - | - | - | - | + |
| Hemolysis | α | α | α | γ | γ | γ | γ | α | α | γ | γ | γ | γ |
| **Growth in:** |  | | | | | | | | | | | | |
| L-Arabinose | + | + | + | + | + | + | + | + | + | + | + | + | + |
| D-Galactose | + | + | + | + | + | + | + | + | + | + (-1) | + | + | + |
| D-Maltose | + | + | + | + | + | + | + | + | + | + | + | + | + |
| D-Mannitol | + | + | + | + | + | + | + | + | + | + | + | + | - |
| D-Melibiose | + | + | + | + | + | + | + | + | + | + | + | + | - |
| D-Raffinose | + | + | + | + | + | + | + | + | + | + | + | + | - |
| L-Rhamnose | + | + | + | + | + | + | + | + | + | + | + | + | - |
| D-Ribose | + | + | + | + | + | + | + | + | + | + | + | + | - |
| Salicin | + | + | + | + | + | + | + | + | + | + | + | + | + |
| D-Sorbitol | + | + | + | + | + | + | + | + | + | + | + | + | - |
| D-Sucrose | + | + | + | + | + | + | + | + | + | + | + | + | + |
| D-Trehalose | + | + | + | + | + | + | + | + | + | + | + | + | - |
| D-Xylose | + | + | + | + | + | + | + | + | + | + | + | + | + |
| **VFA from glucose:** |  | | | | | | | | | | | | |
| Acetic acid | + | + | + | + | + | + | + | + | + | + | + | + | + |
| Propionic acid | - | - | + (-1) | + | + (-1) | - | - | - | - (+2) | - | + (-4) | - | + |
| Butyric acid | - | - | - | - | - | - | - | - | - (+2) | - | - (+1) | - | - |
| Isomer of lactic acid | L | L | L | DL | DL | L | DL | L | L | L | D | DL | DL |

+, positive; -, negative. Numbers in parentheses indicate the number of strains showing the reaction.

**Table S3:** Genomic comparison between strain mPRGC5^T^ and related type strains described by ANIb, ANIm, DDH and AAI value. Strain 1, mPRGC5 ^T^; 2, *S. caprae* JCM 33725^T^; 3, *S. ruminantium* subsp. *lacticlytica* JCM 6582 ^T^; 4, *S. ruminantium* subsp. *ruminantium* DSM 2150 ^T^.

| Strain | ANIb (%) | ANIm (%) | DDH (%) | AAI (%) |
| --- | --- | --- | --- | --- |
| 1 | * | * | * | * |
| 2 | 79.43 | 86.17 | 23.5 | 82.6 |
| 3 | 73.80 | 83.99 | 20.4 | 73.6 |
| 4 | 74.25 | 84.47 | 21.2 | 73.3 |

**Table S4:** Biochemical characteristics of mPRGC5^T^ and its type strains.

| **Items** | **mPRGC5^T^** | **JCM 33725^T^** | **JCM 6582^T^** | **DSM 2150^T^** |
| --- | --- | --- | --- | --- |
| Nitrate reduction | + | + | + | + |
| Catalase test | - | - | - | - |
| Indole production | - | - | - | - |
| H_2_S production | - | - | - | - |
| Voges-Proskauer | - | - | - | - |
| Methyl red test | + | + | + | + |
| Gelatin liquefaction | - | - | - | - |
| Starch hydrolysis | + | - | + | - |
| Casein hydrolysis | - | - | - | - |

| **Acid production from** |  |  |  |  |
| --- | --- | --- | --- | --- |
| Glucose | **+** | **+** | + | + |
| Amidon (starch) | + | - | + | - |
| Amygdalin | - | w | - | - |
| D-Adonitol | - | - | - | w |
| L-Arabinose | + | + | + | w |
| D-Arabitol | - | - | - | w |
| L-Arabitol | - | - | - | - |
| D-Celiobiose | + | + | - | + |
| Dulcitol | - | - | - | - |
| Ecculin ferric citrate | - | - | - | - |
| Erythritol | - | - | - | w |
| Fructose | + | + | + | + |
| D-Galactose | + | + | + | + |
| Glycerol | - | - | - | w |
| Inositol | + | - | - | + |
| Inulin | - | - | - | w |
| D-Lactose (bovine origin) | + | + | + | + |
| D-Maltose | + | + | + | + |
| D-Mannitol | - | + | w | + |
| D-Mannose | + | + | w | + |
| D-Melezitose | - | - | - | w |
| D-Melibiose | - | + | + | + |
| D-Raffinose | - | + | + | + |
| L-Rhamnose | - | - | + | + |
| D-Ribose | - | - | w | + |
| D-Saccharose (sucrose) | + | + | + | + |
| Salicin | + | + | w | + |
| D-Sorbitol | - | - | - | w |
| L-Sorbose | - | - | - | - |
| D-Tagatose | - | - | - | w |
| D-Trehalose | - | - | - | + |
| Xylitol | - | - | - | w |
| D-Xylose | + | + | - | w |

+, positive; w, weakly positive; -, negative.

**Table S5:** The survival rate in gastrointestinal tract of strain MP3 after incubation for 3 hour in various acidic and bile concentrations. Data reported as mean ± SD.

| **Items** | **Results** |
| --- | --- |
| **Number of viable cell, log CFU/mL** |  |
| MRS medium | 8.95 ± 0.04 |
| pH 2 | 3.99 ± 0.18 |
| pH 3 | 5.70 ± 0.58 |
| 0.3% bile | 8.39 ± 0.04 |
| 1.0% bile | 7.98 ± 0.18 |
| **Survival rate, %** |  |
| pH 2 | 56.00 |
| pH 3 | 78.59 |
| 0.3% bile | 90.63 |
| 1.0% bile | 86.73 |

**Table S6:** The putative probiotic gene annotation of *Ligilactobacillus salivarius* MP3, *Lacticaseibacillus rhamnosus* GG ATCC53103^T^ and *Ligilactobacillus salivarius* CGMCC20700.

| Putative function | Genes | Gene product | MP3 | GG | CGMCC20700 |
| --- | --- | --- | --- | --- | --- |
| Modulation of immune system/ Acid stress | *clpB* | Potential immunogenic proteins | + | + | + |
|  | *IspA* | Lipoprotein signal peptidase | + | - | + |
|  | *tuf* | Elongation factor Tu | + | + | + |
| Nutritional synthesis and several essential process | *ccpA* | Catabolite control protein A | + | - | + |
| Cholesterol-lowering effect/ Bile resistance | *bsh* | Choloylglycine hydrolase | + | + | + |
| Adhesion or interaction with the host | *srtA* | Class A sortase | + | + | + |
|  | *dltD* | D-Alanyl-lipoteichoic acid biosynthesis protein DltD | + | + | + |
|  | *dltA* | D-Alanylation of LTA | + | + | + |
|  | *Mub* | Mucus-binding protein | - | - | - |
|  | *glnH1* | Glutamine ABC transporter substrate-binding protein | + (*glnH*) | + | - |
|  | *lspA* | Lipoprotein signal peptidase | + | - | + |
|  | *tuf* | Elongation factor Tu | + | + | + |
|  | *mtsA* | Manganese ABC transporter substrate-binding protein | - | + | - |
|  | *eno2* | Enolase 2 | - | - | - |
|  | *gap* | Type I glyceraldehyde-3-phosphate dehydrogenase | + | + | + |
|  | *groS* | Co-chaperonin GroES | + | + | + |
|  | *groL* | Chaperonin GroEL | + | + | + |
|  | *glnA* | Glutamine synthetase | + | - | + |
|  | *gpi* | Glucose-6-phosphate isomerase | + | + | + |
| Acid stress | *atpC* | ATP synthase subunit epsilon | + | + | + |
|  | *atpD* | ATP synthase subunit beta | + | + | + |
|  | *atpA* | ATP synthase subunit alpha | + | + | + |
|  | *atpG* | ATP synthase subunit gamma | + | + | + |
|  | *atpH* | ATP synthase subunit delta | + | + | + |
|  | *atpF* | ATP synthase subunit B | + | + | + |
|  | *atpB* | ATP synthase subunit A | + | + | + |
|  | *atpE* | ATP synthase subunit C | + | + | + |
|  | *recA* | Protein RecA (recombinase A) | + | + | + |
|  | *relA* | GTP pyrophosphokinase | + | - | + |
|  | *groS* | Co-chaperonin GroES | + | + | + |
|  | *groL* | Chaperonin GroEL | + | + | + |
|  | *aspS* | Aspartate-tRNA ligase | + | + | + |
| Acid stress/Bile resistance | *gpmA1* | 2,3-Bisphosphoglycerate-dependent phosphoglycerate mutase 1 | + | + | + |
|  | *gpmA2* | 2,3-bisphosphoglycerate-dependent phosphoglycerate mutase 2 | + | + | + |
|  | *dnaK* | Chaperone protein DnaK | + | + | + |
|  | *dnaJ* | Chaperone protein DnaJ | + | + | + |
|  | *glmU* | Bifunctional UDP-N acetylglucosamine diphosphorylase/glucosamine phosphate | + | + | + |
|  | *luxS* | S-Ribosylhomocysteine lyase | + | + | + |
|  | *gadB* | Glutamate decarboxylase; GABA transporter | - | - | - |
| Bile resistance | *nagB* | Glucosamine-6-phosphate deaminase | + | + | + |
|  | *pyrG* | CTP synthase | + | + | + |
|  | *argS* | Arginine-tRNA ligase | + | + | + |
|  | *rpsC* | 30S Ribosomal protein S3 | + | + | + |
|  | *rpsE* | 30S Ribosomal protein S5 | + | + | + |
|  | *rplD* | 50S Ribosomal protein L4 | + | + | + |
|  | *LSL_1568* | Beta-N-Acetylhexosaminidase | - | - | - |
|  | *rfa* | Glycosyltransferase | + | + | + |
|  | *LSL_1716* | PTS mannose transporter subunit IIB | + | - | + |
|  | *LSL_1715* | PTS sugar transporter subunit IIC | + | + | + |
|  | *LSL_1714* | PTS N-Acetylglucosamine transporter subunit IIABC | + | - | - |
|  | *LSL_1713* | PTS N-Acetylglucosamine transporter subunit IIBC | + | - | - |
|  | *LSL_1712* | Alpha-glucosidase | - | + | - |
|  | *LSL_1711* | LacI family DNA-binding transcriptional regulator | + | + | + |
|  | *LSL_1709* | Type 1 glutamine amido-transferase | + | + | + |
|  | *LSL_1708* | ArsR family transcriptional regulator | + | + | + |

+, detected; -, no detection.

**Table S7:** The results of pathogenic prediction, prophage detection and antibiotic resistance genes (ARGs) analysis from genome web-based tools (CARD, CGE-PathogenFinder, PlasmidFinder, ResFinder and PHASTER).

| Attribute/Strain | *L. salivarius* MP3 | *L. rhamnosus GG*  ATCC 53103^T^ | *L. salivarius*  CGMCC 20700 |
| --- | --- | --- | --- |
| Probability of being a human pathogen | 0.165 | 0.198 | 0.158 |
| Input proteome coverage (%) | 0.72 | 40.39 | 0.71 |
| Matched pathogenic families | 0 | 0 | 0 |
| Matched not pathogenic families | 13 | 1141 | 11 |
| Conclusion | Non-human pathogen | Non-human pathogen | Non-human pathogen |
| Number of plasmids | 0 | 0 | 0 |
| Number of phages | 5 | 4 | 1 |
| Antibiotic resistance genes (ARGs) |  |  |  |
| CARD: |  |  |  |
| - Perfect hits | 0 | 0 | 0 |
| - Strict hits | 4 (*VanT, tet*(M), *fex*B, *Erm*C) | 0 | 1 (*Van*T) |
| - Loose hits | 0 | 0 | 0 |
| ResFinder: | *tet*(M), *fex*B, *Erm*C | No resistance | No resistance |

**Table S8:** Genomic features of *Ligilactobacillus salivarius* MP3, *Lacticaseibacillus rhamnosus* GG ATCC 53103 and *Ligilactobacillus salivarius* CGMCC20700.

| Attribute | MP3 | LGG | CGMCC20700 |
| --- | --- | --- | --- |
| Source | Goat rumen fluid | Fecal sample of healthy human | Intestinal mucosa of Yunnan chicken |
| Accession no. | JANFQI000000000 | FM179322 | CP101685 |
| Genome size (bp) | 1,876,541 | 3,010,111 | 1,737,577 |
| Plasmids | 0 | 0 | 2 |
| Genome qualities: |  |  |  |
| - Genome quality | Good | Good | Good |
| - Completeness (%) | 96.4 | 99.46 | 99.48 |
| - Coarse consistency | 99.9 | 99.4 | 99.8 |
| - Fine consistency | 99.8 | 98.3 | 99.6 |
| G+C content (%) | 32.6 | 46.7 | 33.1 |
| Genome coverage | 178x | 1953.0x | 674.0x |
| N50 | 171,507 | 3,005,051 | 1,737,577 |
| L50 | 4 | 1 | 1 |
| No. of contig | 48 | 1 | 1 |
| No. of subsystem | 213 | 231 | 199 |
| No. of coding sequences | 1853 | 2995 | 1565 |
| No. of RNA | 52 | 74 | 100 |
| No. of CRISPRS | 1 | 1 | 2 |
